# Transport and Interfacial Injection of d-Band Hot Holes Control Plasmonic Chemistry

**DOI:** 10.1021/acsenergylett.3c01505

**Published:** 2023-09-19

**Authors:** Fatemeh Kiani, Alan R. Bowman, Milad Sabzehparvar, Can O. Karaman, Ravishankar Sundararaman, Giulia Tagliabue

**Affiliations:** † Laboratory of Nanoscience for Energy Technologies (LNET), STI, École Polytechnique Fédérale de Lausanne, 1015 Lausanne, Switzerland; ‡ Department of Materials Science & Engineering, Rensselaer Polytechnic Institute, 110 Eighth Street, Troy, New York 12180, United States

## Abstract

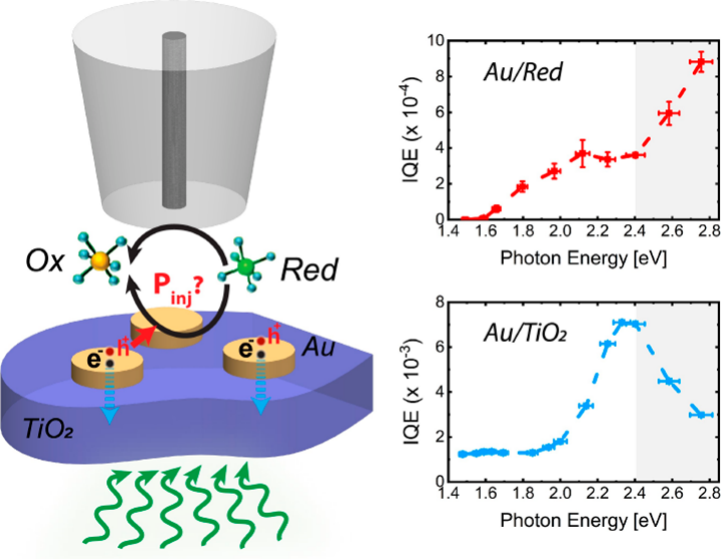

Harnessing nonequilibrium hot carriers from plasmonic metal nanostructures constitutes a vibrant research field with the potential to control photochemical reactions, particularly for solar fuel generation. However, a comprehensive understanding of the interplay of plasmonic hot-carrier-driven processes in metal/semiconducting heterostructures has remained elusive. In this work, we reveal the complex interdependence among plasmon excitation, hot-carrier generation, transport, and interfacial collection in plasmonic photocatalytic devices, uniquely determining the charge injection efficiency at the solid/liquid interface. Measuring the internal quantum efficiency of ultrathin (14–33 nm) single-crystalline plasmonic gold (Au) nanoantenna arrays on titanium dioxide substrates, we find that the performance of the device is limited by hot hole collection at the metal/electrolyte interface. Our solid- and liquid-state experimental approach, combined with *ab initio* simulations, demonstrates more efficient collection of high-energy d-band holes traveling in the [111] orientation, enhancing oxidation reactions on {111} surfaces. These findings establish new guidelines for optimizing plasmonic photocatalytic systems and optoelectronic devices.

Prompt collection of photoexcited hot carriers in plasmonic metal nanostructures offers substantial promises for the development of applications such as tunable photodetection^1−3^ and selective photocatalysis.^4−7^ Practical realizations of hot-carrier devices thus require a full understanding of plasmonic hot-carrier-driven processes, including plasmon excitation (optical response), hot-carrier generation, carrier transport, and collection at solid/solid or solid/liquid interfaces. To date, despite significant experimental and theoretical investigations on plasmon excitation and hot-carrier generation, electronic processes, i.e., transport and collection, have been less considered. Specifically, works have been able to unravel in detail the picture of the hot electron or hot hole collection at the solid/solid interface,^1,2,8−10^ but fewer studies have tried to resolve how the charge carrier collection occurs at the solid/liquid interface.^11−13^ In fact, the majority of the focus has been on analyzing the external quantum efficiency (EQE) and the photoinduced activity of photocatalysts based on the plasmon resonance absorption^14−16^ or on tracking molecular transformations via Raman spectroscopy.^17−19^ Carriers generated by plasmon decay impinge upon the surface of a plasmonic nanostructure to be collected, either ballistically or after scattering against other carriers, phonons, or defects in the metal. These ultrafast (a few tens of femtoseconds to picoseconds) scattering processes thermalize the carriers and bring their energy distribution closer to the Fermi level of the metal.^20^ However, plasmonic hot-carrier applications require high-energy electrons or holes to efficiently drive the ensuing processes.

Plasmon-driven photocatalytic devices typically employ metallic nanoantennas/semiconductor heterostructures due to their efficient hot-carrier separation. As a result, hot-carrier-driven processes typically involve the complex interplay of carrier collection at both metal/semiconductor and metal/electrolyte interfaces. To gain a complete system understanding, the geometry of the nanostructures and their absorption properties need to be precisely controlled. In particular, polycrystalline metal structures or films that have been used in almost all of the plasmonic metal/semiconductor devices are not ideal for fundamental studies. Instead, single-crystalline nanoparticles or metal films, e.g. gold microflakes,^21,22^ can be leveraged to obtain high-quality plasmonic nanoantenna (arrays) with unique optical properties^23−25^ and well-defined crystallographic surfaces, which exhibit distinct catalytic properties^26,27^ as well as distinct hot-carrier transport properties.^28^ Additionally, experimental quantification of the internal quantum efficiency (IQE) of hot-carrier collection at each interface is critical to clarify the role of interfaces and their interplay and to further elucidate the potential opportunities and limitations of hot-carrier collection in these devices. Moreover, an appropriate model for hot-carrier injection across metal/electrolyte interfaces has remained elusive. Overall, the lack of comprehensive experimental approach, a theoretical model, and a highly controlled nanostructure system have prevented an in-depth investigation on the hot-carrier transport and collection processes occurring within hot-carrier-driven photocatalytic systems.

In this work, we implement a combination of solid-state photocurrent and liquid-state photoelectrochemical measurements to simultaneously study the transport and collection of hot carriers across the metal/semiconductor and metal/electrolyte interfaces (Figure 1a). Uniquely, we leverage highly controlled single-crystalline plasmonic gold (Au) nanoantenna arrays with precisely defined thicknesses on a TiO_2_ semiconducting substrate, and we locally probe hot-hole-driven photo-oxidation of a redox molecule at different excitation wavelengths (1.4–2.8 eV) using light-assisted scanning electrochemical microscopy (photo-SECM). Our analysis reveals that the IQE of the photoanode device, in which both hot electrons and hot holes are collected, is controlled and limited by the hot-hole collection. Furthermore, through a combination of experimentally determined IQEs and *ab initio* predictions of hot-carrier generation and transport, we developed a hot-hole-injection probability model. This shows that d-band high-energy holes have maximum extraction probability, particularly from the top {111} facet. Additionally, we find that the thickness plays an important role and that the thinnest (16 nm) nanoantennas are the most efficient for the ballistic collection of d-band hot holes. Overall, our highly controlled experimental approach allows quantitative comparison with optical modeling and theoretical calculations, enabling the deconvolution of separate optical and electronic contributions in hot-carrier-driven photocatalytic systems. This information and methodology can thus play a critical role toward better design and optimization of plasmonic catalysts.

**Figure 1:**
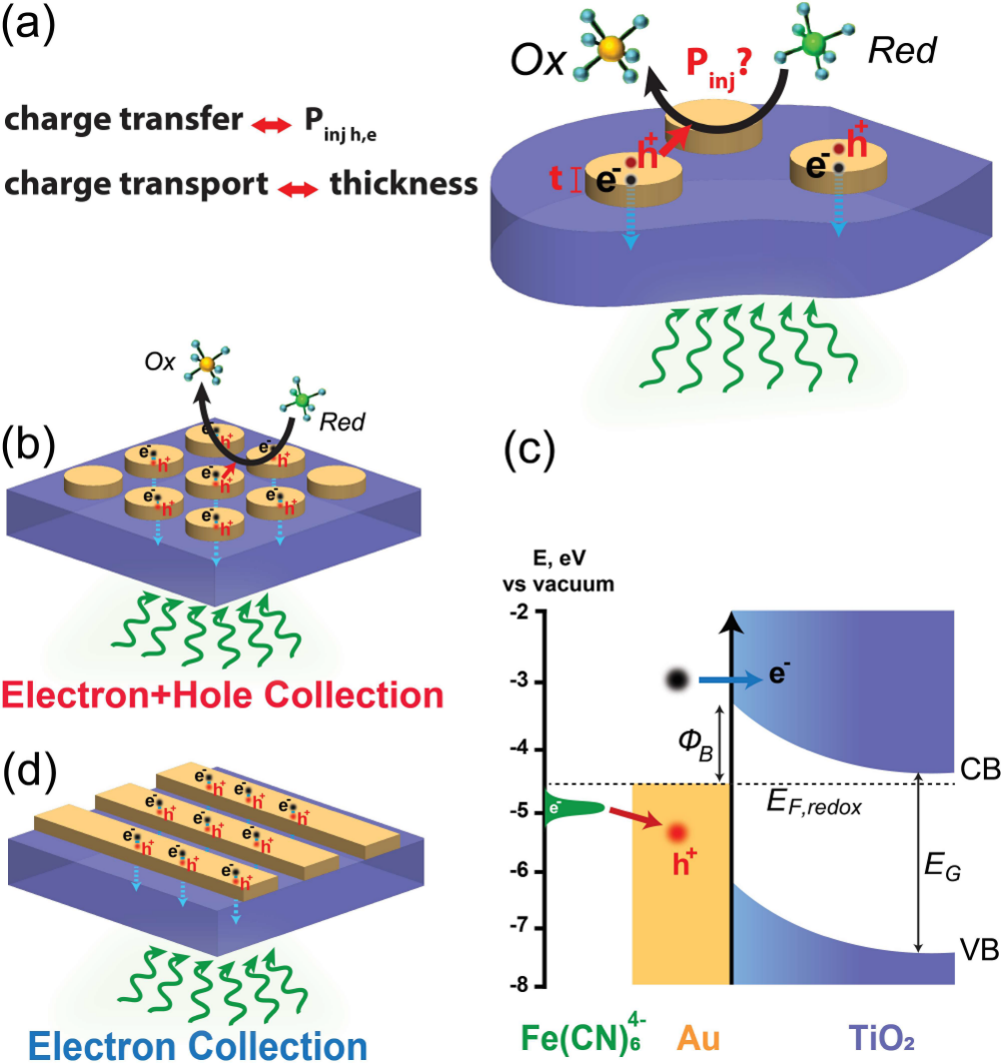
Schematic of interfacial hot carrier collection in plasmonic metal/semiconductor heterostructure devices. (a) Transport and injection of hot carriers across the metal/semiconductor and metal/electrolyte interfaces. (b) Au NDs/TiO_2_ photoanode in contact with a reductant (*Red*) molecule and (c) band alignment showing hot-electron and hot-hole collection across the Au/TiO_2_ and Au/Fe(CN)_6_^4–^ interfaces, respectively. (d) Au stripes/TiO_2_ photodiode geometry that operates via hot-electron collection.

We fabricated plasmonic photocatalytic devices (photoanode) consisting of an array of Au nanodisks (Au NDs) with sizes on the order of tens of nanometers on top of a TiO_2_/ITO film on a fused silica substrates (Figure 1b). Uniquely, our nanoantennas are made from single-crystalline Au microflakes (SC Au MFs)^21^ (see Methods in Supporting Information S1.1) to exclude the influence of grain boundaries and thus reduce Ohmic losses. Importantly, their atomically smooth and well-defined {111} crystallographic surfaces allow us to deconvolute the effect of different crystallographic facets from other effects in plasmonic photocatalysis. We use TiO_2_ because of its large optical band gap (EG ≈ 3.2 eV^29^), preventing visible-light absorption within the TiO_2_ film, and we note it forms a Schottky barrier (Φ_B_) across the Au/TiO_2_ heterojunction, enabling hot-electron collection (Figure 1c). The Au NDs are in contact with an electrolyte containing a reversible redox molecule, Fe(CN)_6_^4–^ (ferrocyanide, the reduced form, *Red*), which enables hot-hole collection through a photoelectrochemical oxidation reaction (Figure 1a,c). The oxidaton of this molecule proceeds via a one-electron-transfer, outer-sphere mechanism with fast kinetics.^30^ Moreover, the chosen molecule does not absorb visible light (see Figure S1). For complementary solid-state studies, we also work on a plasmonic photodetector device (Schottky photodiode) consisting of an array of SC Au stripes on the same TiO_2_/ITO film (see Supporting Information S1.1), providing an ideal experimental platform for studying the hot-electron collection from the same metal with the same semiconductor (Figure 1d). The stripe geometry is dictated by the need for a direct electrical contact to the plasmonic nanoantennas. Since the bottom interface as well as the edges are similar in both disk and stripe heterostructures, and considering that the spatial electric field profile is comparable at this interface for both systems (Figure S6), we expect that electron injection, which is controlled by the properties of the interface,^31^ is identical in both devices. The nanoantennas were fabricated from Au MFs with varying thicknesses of 14–33 nm to study the effect of nanoantenna thickness on hot carrier generation, transport, and collection processes within our device.

Photo-SECM is a unique technique for ultrasensitive^32^ and fast detection^33^ of tiny photochemical reactions on small-size nanostructures.^34−36^ In contrast to standard photoelectrochemical systems, it employs a biased electrochemical probe, i.e., a Pt ultramicroelectrode (Pt UME) tip, positioned very close to a photoilluminated nanostructure. This allows for the detection of extremely weak photochemical reactions occurring locally on the substrate surface and at power intensities as small as ∼1 W/cm^2^, where photothermal heating effects are negligible.^14,37,38^ For photoelectrochemical experiments, we performed a series of photo-SECM measurements on Au ND arrays with disk thicknesses of 16, 25, and 33 nm and average diameters of 68, 80, and 80 nm, respectively. We present the implemented photo-SECM approach in Figure 2a. SEM image in Figure 2b shows one of our fabricated ND arrays for a 25 nm thick Au MF with a lateral size of 140 μm on a TiO_2_/ITO-coated fused silica substrate. The SEM and AFM images in Figure 2c,d show a magnified view of the fabricated ND structure. SEM and AFM images of the other fabricated ND array structures are shown in Figure S3. Thanks to the single crystallinity of the flakes, the fabricated nanoantennas exhibit an exact shape and size and ultrasmooth surfaces.

**Figure 2:**
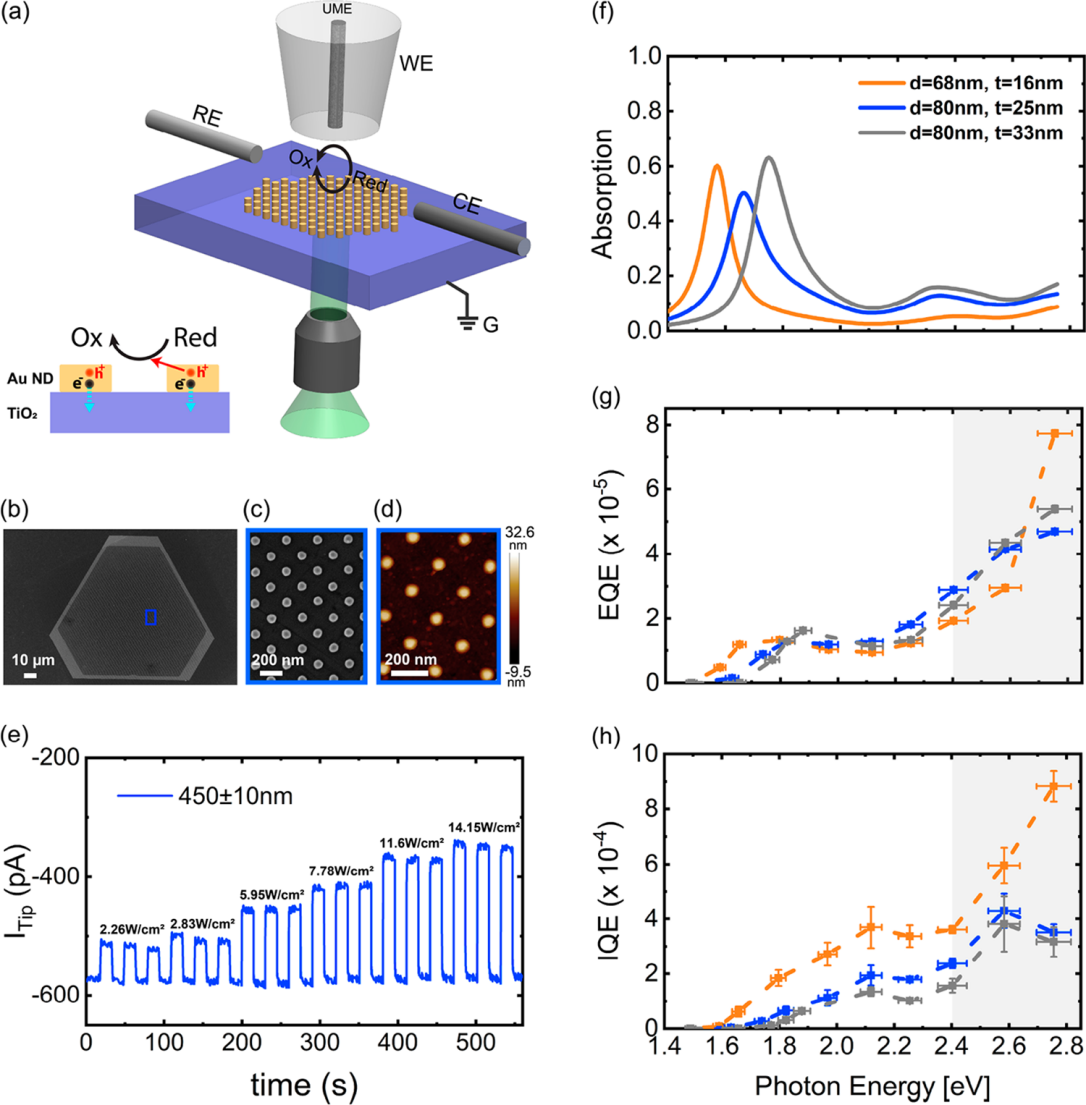
Liquid-state photoelectrochemical measurement results. (a) Schematic of the designed plasmonic heterostructure and photo-SECM configuration in competition experiment mode. A fabricated Au ND array from a SC Au MF on TiO_2_/ITO substrate is in contact with an electrolyte contacting 4 mM Fe(CN)_6_^4–^ (*Red*) and 0.25 M KCl. A 1.2 μm radius Pt ultramicro electrode (UME) tip is positioned 2.5 μm away from the substrate. The UME tip is biased at 0.4 V vs Ag/AgCl (reference electrode, RE), and the substrate is at open circuit and is grounded. Light is incident on the plasmonic Au ND array from the bottom. The same oxidation reaction occurs at the tip electrode and substrate surface. The current is measured through the tip electrode (working electrode, WE). A Pt wire is used as a counter electrode (CE) to complete the circuit. The side-view schematic in (a) illustrates the direction of carrier transfer at interfaces. (b) SEM image of an entirely patterned 25 nm thick Au MF. (c) Higher magnification SEM and (d) AFM images of the fabricated Au NDs array from the Au MF in (b). The average ND diameter and thickness are 80 and 25 nm, respectively. The array periodicity is 200 nm. (e) Time trace of tip current (*i*_Tip_) obtained from the 1.2 μm radius Pt UME upon illumination of the Au ND array in (b) with an excitation wavelength of 450 ± 10 nm at different light intensities. (f) Simulated absorption spectra of the fabricated heterostructures having different Au ND thicknesses of 16, 25, and 33 nm exhibiting resonance peaks at 1.57, 1.66, and 1.75 eV, respectively. (g) Experimentally determined EQE and (h) IQE spectra for the same heterostructures. The gray shaded areas depict the purely interband region, and the dashed lines are a guide to the eye in (g) and (h).

We determined absorption spectra of the structures experimentally and by simulation under front illumination in ambient air conditions (see Supporting Information S.1.2,4 and Figure S4). Measured absorptions are in good agreement with numerical simulations (Figure S4, dashed lines). To determine the absorption spectra with the illumination condition required for photoelectrochemical experiments, we implemented simulations for an aqueous medium and back-illumination condition (see Supporting Information S1.4). Calculated absorption spectra are plotted in Figure 2f. All the Au ND array heterostructures show a dipole plasmon resonance mode in the intraband region (1.57–1.75 eV), which enables us to disentangle plasmon absorption and interband excitation effects. Disentangling these two effects was challenging in previous studies, as the resonance of the structure overlapped with the interband regime.^14−16^

For photoelectrochemical experiments, we used an aqueous electrolyte solution containing 4 mM Fe(CN)_6_^4–^ and 0.25 M KCl as the supporting electrolyte and positioned a 2.4 μm Pt UME tip 2.5 μm away from the substrate. We performed photo-SECM measurements in competition mode, where an oxidation reaction proceeds at both the UME tip and the substrate (Figure 2a). During the competition SECM experiment, we applied a potential of 0.4 V vs Ag/AgCl at the Pt UME tip corresponding to electro-oxidation of Fe(CN)_6_^4–^ at a diffusion-controlled rate (see Figure S2b) while the substrate was illuminated from the bottom with a tunable, monochromatic light at different photon energies (see Supporting Information S1.3). The substrate was under open-circuit conditions and grounded to take away the accumulated electrons in the ITO layer (Figure 2a). We focused the laser beam on the Au NDs with a spot diameter of 30 μm to drive the photo-oxidation reaction only at the illuminated area of the substrate upon hot carrier generation and hot hole transfer at the Au/electrolyte interface. We measured the current through the tip as a function of excitation power to monitor changes in the local concentration of Fe(CN)_6_^4–^ and thus the photoelectrochemical dynamics at the plasmonic substrate. Figure 2e shows the time trace of the tip current (*i*_Tip_) upon illumination of a 25 nm thick ND array at an excitation wavelength of 450 ± 10 nm, where the intensity was modulated up to ∼14 W/cm^2^. To show the repeatability of the results, we performed the measurements three times at each illumination intensity. We note that once the light is turned off, *i*_Tip_ returns to the baseline current (*i*_Tip,dark_), indicating the rapid diffusion and charge transfer of the redox molecules within the tip–substrate gap that enables the achievement of a steady-state response easily. We observe a linear decrease in the magnitude of *i*_Tip_ relative to the dark current (*i*_Tip_/*i*_Tip,dark_) by increasing the excitation intensity (Figure S10b). This decreasing trend indicates that the local concentration of Fe(CN)_6_^4–^ at the tip–substrate gap decreases due to a hole-driven oxidation reaction at the plasmonic substrate, which gets enhanced in kinetics by illumination intensity. To confirm that the observed enhanced photo-oxidation at the Au NDs originates from the hot-carrier generation and collection rather than photothermal heating effects in the range of the studied excitation intensities, we implemented a SECM approach^37,38^ for probing the photothermal heating at a plasmonic substrate (see Supporting Information S.4). Local heating at a plasmonic substrate results in enhanced mass transfer rates of the redox molecules, as well as a shift in the formal potential of a redox couple.^37^ Our SECM measurements showed no sign of the local heating effects on the 25 nm thick Au NDs array under illumination at its plasmonic resonance and excitation intensities up to 330 W/cm^2^ (Figure S7). We have also theoretically confirmed that the temperature increase remains insignificant in our experiments (Supporting Information S.5, Figure S8).

To investigate the wavelength dependence of the photochemical oxidation rate of Fe(CN)_6_^4–^, we studied a broad excitation wavelength range of 450–832 nm covering the entire intra- and interband transition regimes for hot-carrier generation in Au NDs. The measured *i*_Tip_/*i*_Tip,dark_ responses under different excitation intensities at each wavelength are shown in Figure S10b. We implemented a diffusion model^14^ to simulate calibration curves correlating *i*_Tip_/*i*_Tip,dark_, substrate photocurrent (*i*_sub,photo_), and the reaction rate constant (*K*_eff_) of the photo-oxidation reaction of Fe(CN)_6_^4–^ at the substrate (see Supporting Information S.6Figures S9 and S10). The substrate photocurrents were extracted using the calibration curve obtained by the diffusion model (Figure S10c) and the measured data of *i*_Tip_/*i*_Tip,dark_ under different illumination intensities (Figure S10b) for each excitation wavelength and plotted as a function of power in Figure S10d. As a control experiment, the same SECM measurement was performed on a bare TiO_2_/ITO substrate in the absence of Au NDs. A slightly enhanced photoinduced *i*_Tip_ was observed at the shorter wavelength region by increasing the excitation intensity (Figure S11). To exclude the contribution from the TiO_2_ substrate, the TiO_2_/ITO photocurrent was subtracted from the Au/TiO_2_/ITO photocurrent. Therefore, the substrate photocurrent plots in Figure S10d after the subtraction correspond solely to the hot-hole-driven photo-oxidation reaction at Au ND surfaces. The external quantum efficiency (EQE) of the photoelectrochemical reaction is determined from the slope of the linear fit to *i*_sub,photo_ vs illumination power curves for each excitation wavelength using eq S26 (see Supporting Information S.6). The linear relationship of *i*_sub,photo_ versus power shows different slopes, clearly reflecting that the quantum efficiencies of the hot-hole-driven oxidation reaction are wavelength-dependent. The measured absorption allows us to calculate the IQE, defined as the probability of a chemical reaction per absorption photon. The obtained EQE and IQE spectra are plotted in Figure 2.g,h for all of the ND arrays (see Figure S12 for normalized EQE and IQE spectra). We observe a common feature in all EQE spectra: a peak associated with the characteristic peak plasmon absorption energy in each structure. Conversely, the IQEs are not purely monotonic and interestingly all the three IQE curves exhibit an intermediate peak at around 585 nm (2.12 eV) and the maximum efficiency was obtained in the interband region (>2.4 eV, gray area in Figure 2h), as observed in previous studies.^11−13^ Comparing the thickness-dependent IQE spectra, we find a significant difference between the 16 nm and thicker ND structures, in particular, in the interband region. The IQE of the 16 nm ND structure increases monotonically from 2.4 to 2.8 eV, while the IQE of 25 and 33 nm ND structures rises up to a peak at 2.6 eV and then drops at higher photon energies of up to 2.8 eV. This is possibly because of an increased charge recombination at the Au/TiO_2_ interface due to the concurrent charge generation in TiO_2_ at higher photon energies, as also observed in previous studies.^39,40^ Nonetheless, this interfacial recombination does not impact the performance of the 16 nm thick NDs. Overall, these observations suggest that decreasing the nanoantenna thickness does alter the hot-carrier-driven processes across the interfaces. In particular, as we will show quantitatively later, they indicate that hot-hole transport plays a critical role in determining the device performance. Yet, as both hot electrons and hot holes are collected at different surfaces, it is first necessary to understand the role of the metal/semiconductor interface and the hot-electron collection at this boundary.

To study hot-electron collection in the solid state, we fabricated a plasmonic Schottky photodiode device consisting of an array of Au stripes with a thickness of 14 nm, close to the thickness of the best-performing ND device on the same TiO_2_/ITO film. We present the implemented solid-state approach in Figure 3a. The SEM image in Figure 3b shows a fabricated 30 × 30 μm^2^ stripe array. The magnified SEM and AFM images (Figure 3c,d) show the fabricated stripe structure with a width of 70 nm and a periodicity of 230 nm. From Figure 3b we observe that the stripe array is connected to the Au flake body, which can enable photocurrent measurements by electrically contacting microprobes on a nonpatterned flake area and on a sputtered Ohmic contact to TiO_2_. The photocurrent can be collected while illuminating the sample through the bottom with a tunable, monochromatic light (see the schematic in Figure 3a and Supporting Information S1.3). Figure 3e shows the time trace of the short-circuit current (*I*_SC_) upon illumination of the device with wavelengths from 450 to 840 nm at different powers. From the *I–V* response shown in Figure 2f, highly rectifying behavior was observed. Fitting this plot to the diode equation^41^ allowed the estimation of a Schottky barrier of ∼1.25 eV across the Au/TiO_2_ heterojunction. We determined the EQE and absorption spectra of the device experimentally by measuring both the wavelength-dependent photocurrent and the transmission and reflection spectra under the same light polarization conditions (see Supporting Information S1.2,3) and plotted them in Figure 3g. A resonance peak at 665 nm (1.85 eV) appears in both spectra. This feature disappears when the light polarization is parallel to the stripes (Figure S5). Such behavior confirms that the photocurrent originates from optical excitation of the dipolar plasmon mode in the nanoantennas.^1^ The observed similar EQE trend with the absorption spectrum indicates that the plasmon excitation manifests itself in an enhanced EQE of the device. Interestingly, consistent with the previous solid-state works,^1,2^ we observed that the IQE spectrum (Figure 3h) exhibits a spectral feature peaking at 550 nm (2.25 eV). The asymmetry between energy distributions for hot carriers generated by interband transitions^20^ (peak of distribution at lower energies close to the Fermi level (*E*_F_) for hot electrons), combined with our Schottky barrier height (1.25 eV), has consequences for hot-electron collection efficiency across the Au/TiO_2_ interface. As a result, we observe an abrupt drop in the solid-state IQE at energies above the interband threshold (gray area in Figure 3h). On the other hand, due to the generation of high-energy d-band holes, there is still the possibility of hole collection at the Au/electrolyte interface, resulting in a continued IQE growth in the entire interband regime for the best performing device (cf. Figure 2h, orange dashed curve, 2.4–2.8 eV). Comparing the liquid-state photoelectrochemical and solid-state photocurrent measurement results, we find different magnitudes and trends between the experimentally obtained IQEs for the two devices. Higher magnitude of the IQE for the hot-electron photodetector device and a very different IQE trend in particular in the interband regime imply that the IQEs of the photoanodes are not limited by the hot-electron collection but rather by the hot-hole collection.

**Figure 3:**
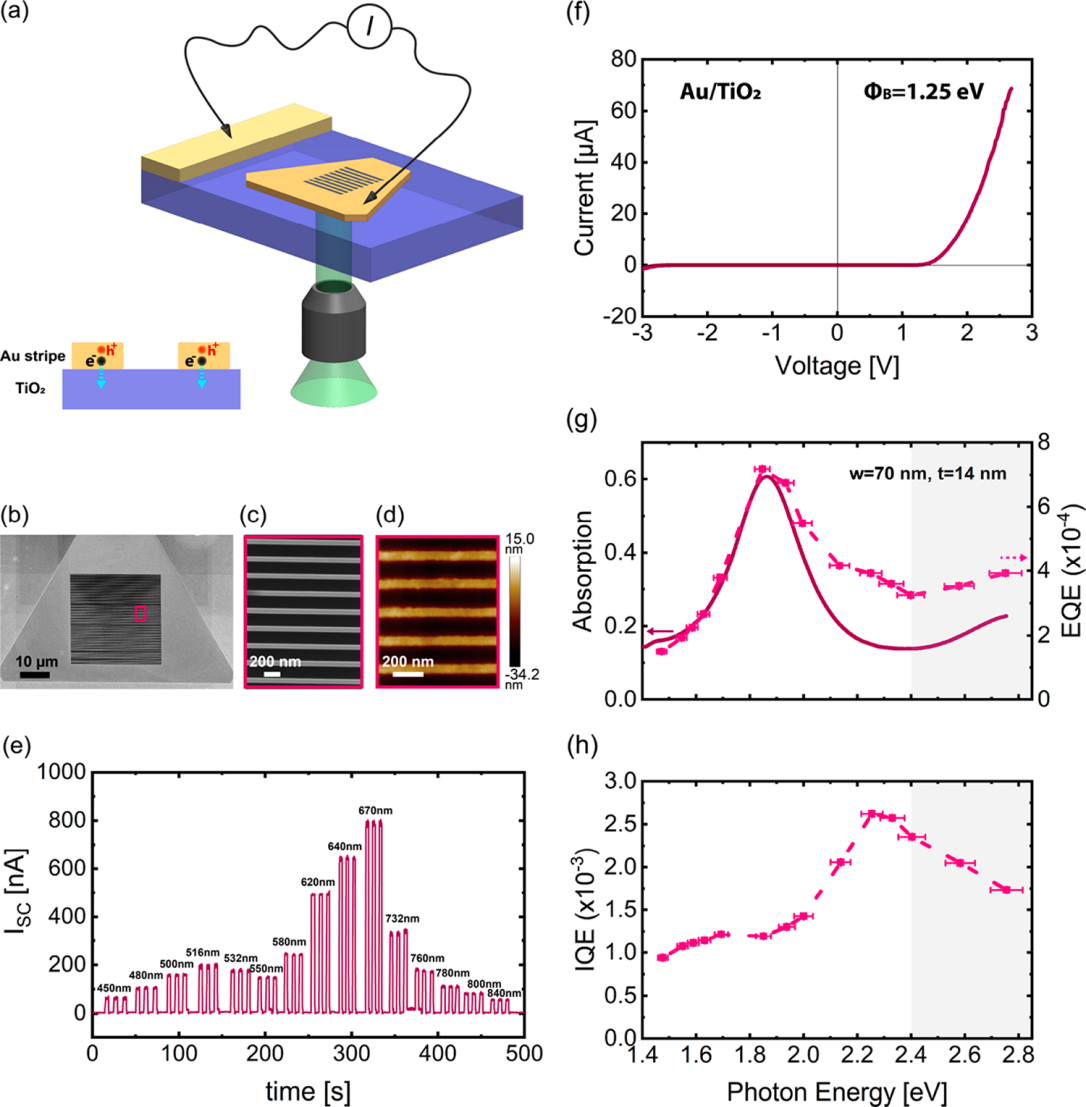
Solid-state photocurrent measurement results. (a) Schematic of the designed plasmonic heterostructure and measurement configuration. A stripe Au pattern is fabricated from a SC Au MF on TiO_2_/ITO substrate together with a 100 nm thick sputtered Au film Ohmic contact. Light is incident on the plasmonic Au stripe array from the bottom, and the photocurrent is collected by two microcontact probes electrically connected to the Au flake and the sputtered Au contact pad. The side-view schematic in (a) illustrates the direction of electron transfer at the Au/TiO_2_ interface. (b) SEM image of a 30 × 30 μm^2^ stripe array from a 14 nm thick Au MF. (c) Higher magnification SEM and (d) AFM images of the fabricated Au stripe array from the Au MF in (b). The average stripe width and thickness are 70 and 14 nm, respectively. The array periodicity is 230 nm. (e) Time trace of the short-circuit current (*I*_SC_) for the fabricated stripe array in (b) upon illumination with excitation wavelengths from 450 ± 10 to 840 ± 10 nm. (f) Measured *I*–*V* plot of the fabricated heterostructure showing a metal–semiconductor Schottky diode behavior across the Au/TiO_2_ interface. A Schottky barrier height of Φ_B_ = 1.25 eV was estimated after fitting these data. (g) EQE (purple dashed curve) and absorption spectra (purple solid curve) of the fabricated heterostructure exhibiting the EQE peak response at the plasmon resonance of the structure at 1.85 eV. (h) Experimentally determined IQE spectrum of the same heterostructure. The gray shaded areas depict the purely interband region, and the dashed lines are a guide to the eye in panels (g) and (h).

To be able to understand how the hot hole collection limits the IQE in the photocatalytic system, we leverage theory^42^ to disentangle the mechanisms controlling the generation, transport, and injection of the hot carriers across the metal/electrolyte and metal/semiconductor interfaces. Figure 4a shows the calculated spatially resolved absorption 2D profiles (i.e. hot-carrier-generation profiles) in Au NDs with thicknesses of 16 and 33 nm on a TiO_2_/ITO/glass substrate at their plasmon resonance (intraband regime) and at photon energy of 2.75 eV (450 nm, interband regime). We observe a uniform hot carrier generation across the whole volume of the 16 nm ND close to the both solid–solid and solid–liquid interfaces while there is a localized generation at the solid–solid interface of the 33 nm ND for both on-resonance and off-resonance excitations. We then employed *ab initio* simulations^42^ to elucidate the impact of carrier transport and separate the contributions from carriers based on the number of times they are scattered before collection (*N*) (see Supporting Information S1.5). The energy-resolved flux, *F*_N_(*E*,ℏω), of hot carriers with energy (*E*) above (hot electrons) and below (hot holes) *E*_F_ that can reach all interfaces was calculated at each photon energy (ℏω). As the fabricated structures are single-crystalline, no orientational averaging was applied on the ballistic hot-carrier fluxes. We calculated the carrier fluxes for {111} top and bottom and {110} side facets^21^ separately. Since the collection of hot holes limits the IQE of the photoanodes, our following discussion will exclusively focus on hot holes. Figure 4b presents a comparison of spatially resolved carrier fluxes of hot holes with energy greater than a threshold (−0.15 or −2 eV) for 16 and 33 nm thick Au NDs at 450 nm and their plasmon resonance wavelength. −0.15 eV is close to the highest occupied molecular orbital (HOMO) level of Fe(CN)_6_^4–^ (−0.19 eV, Supporting Information S.2 and Figure S2), and the −2 eV threshold highlights the available high-energy hot holes. Under on-resonance illumination of the structures in the intraband regime, the maximum flux of the holes that can reach the interface for the 16 and 33 nm NDs are comparable; however, we see a clear difference in their spatial distribution. For the 16 nm ND, the flux is well distributed across the interfaces, while a localized flux is observed on the side surface close to the bottom interface of the thicker ND, as is evident from the corresponding calculated energy-resolved carrier fluxes at different surfaces shown in Figure S13. At energies above the interband threshold, accessible by the 450 nm illumination, the carrier flux becomes more uniform also for the 33 nm ND due to the improved generation profile (Figure 4a at 450 nm). But still, the spatial flux distribution at the top and side surfaces is more intense in the 16 nm ND and it exhibits a higher maximum flux for both energy thresholds compared to the 33 nm ND. Therefore, due to the observed nonuniform carrier generation and flux distribution, as well as the increased distance that holes must travel before reaching the Au/electrolyte interface in the thicker nanoantennas, a slower IQE growth was observed compared to the 16 nm ND (Figure 2h). This is more critical for collecting the high-energy d-band holes, where the mean free path of hot holes decreases to just a few nanometers,^18,40^ leading to a nonmonotonic behavior of thicker ND IQEs upon transition from partially to entirely interband excitation (Figure 2h, 2.1–2.8 eV). Figure 4c maps the mean free path of holes in the top d-band in Au across velocity directions. The unit vector of velocity directions of these holes are distributed nonuniformly across the unit sphere with a much higher probability between the [110] and [111] directions. These most probable velocity directions also have longer mean free paths, indicating that the anisotropy favors hot-hole transport toward the top and sides of our single-crystalline samples (compared to intermediate directions).

**Figure 4:**
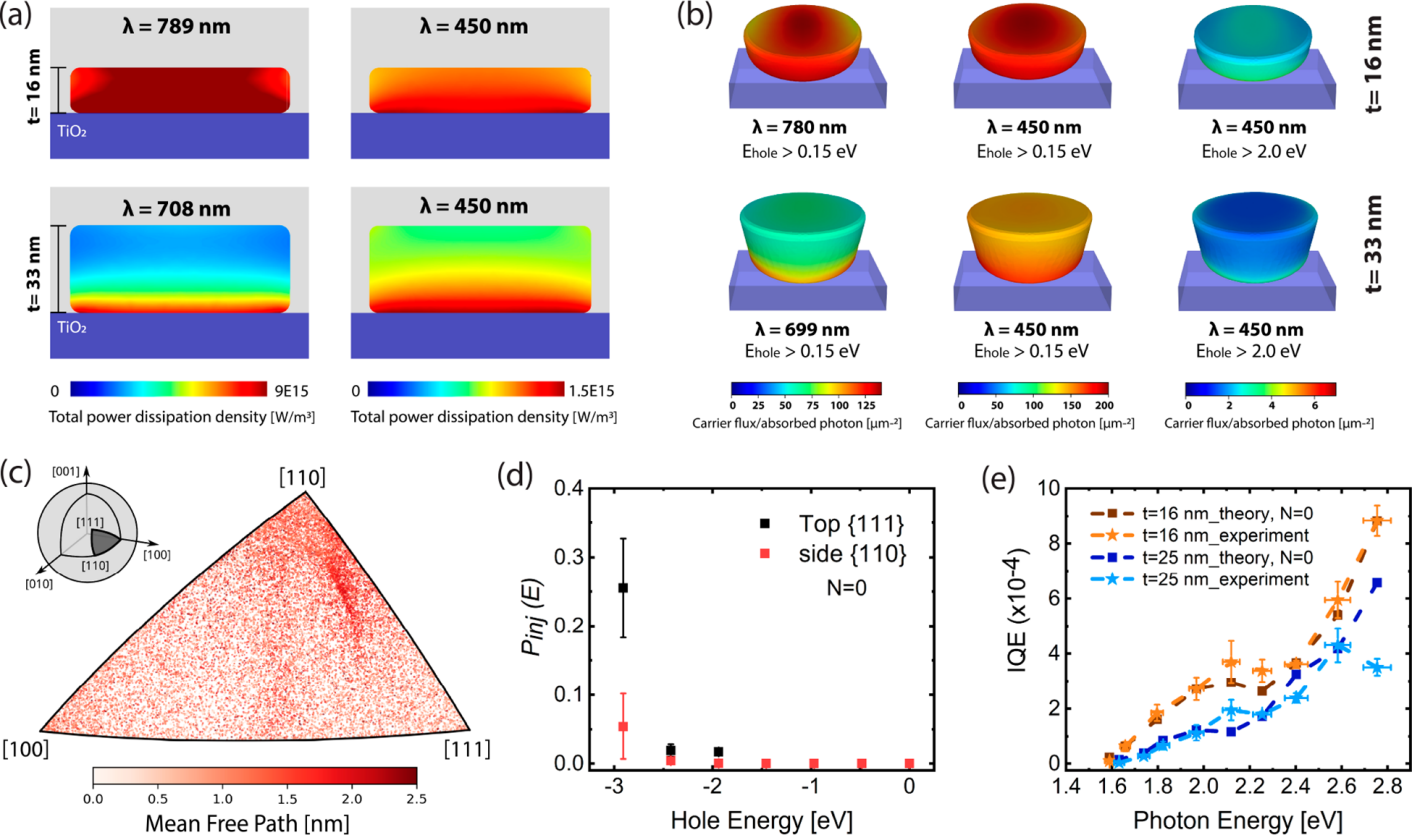
Hot-carrier generation, transport, and injection in plasmonic heterostructure devices. Calculated spatially resolved (a) 2D absorption profiles and (b) hot-hole fluxes reaching the interfaces greater than 0.15 and 2 eV under illumination at 450 nm and the resonance wavelength of the Au NDs having thicknesses of 16 and 33 nm. Fluxes are normalized to the absorbed photon and unit area of the surface. (c) Distribution of velocity unit vectors of holes in the top d-band of Au, colored by mean free path, and shown within the irreducible wedge of the unit sphere under cubic symmetry (bounded by the [100], [110], and [111] crystallographic orientations as vertices). The inset shows the triangular section of the unit sphere with the legends for the velocity directions forming the vertices of this triangle. (d) Injection probability (*P*_inj_) for hot holes collected from the top {111} and side {110} facets. The *P*_inj_ plots are extracted from the fitting approach using the energy-resolved hot-hole fluxes and experimentally determined IQEs of Au ND heterostructures. (e) Calculated IQE spectra based on energy-resolved hot-hole fluxes and estimated *P*_inj_ in (d) for nonscattered carriers (*N* = 0) together with the experimentally determined IQEs for 16 and 33 nm thick Au ND heterostructures. Dashed lines are a guide for the eye.

Finally, we developed an approach to extract the hole injection probability from gold, *P*_inj_(*E*). By assuming that *P*_inj_(*E*) is constant for all samples (as the same surfaces are exposed) and is only a function of the hole energy when a hole reaches a surface (i.e., not a function of incident photon energy), we can state that for the NDs
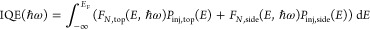
1

Here ℏω is the photon energy, *E* is the energy of the hot holes with respect to *E*_F_, *N* is the number of the scattering events we include in modeling, and top/side subscripts refer to the top/side surfaces of the structure. As we know *F*_*N*_(*E*,ℏω), we can theoretically calculate IQEs based on an assumed *P*_inj_(*E*). We developed a stochastic fitting procedure that varied *P*_inj_(*E*) to minimize the difference between experimental and calculated IQEs (see Supporting Information S1.5 for further details). By performing this fitting procedure hundreds of times, we were able to calculate the average and range of possible *P*_inj_(*E*) values. The resulting *P*_inj_(*E*) from this fitting approach for the top and side surfaces is shown in Figure 4d. No significant difference was obtained in injection probability between nonscattered (Figure 4d, *N* = 0) and scattered (Figure S14, *N* = 4) holes, as the flux distribution of initial and homogenized carriers is the same at energies below −2 eV (Figure S15). We observe the extraction probability approaches an exponentially growing curve for increasing hole energy, with around 5 times higher probability of −2.9 eV holes collected from the top compared to the ones collected from the side surfaces (Figure 4d). A very low upper-bound injection probability of ∼0.1% is obtained for intraband generated holes with an energy of −1.46 eV, although they could have sufficient energy to participate in HOMO electron transfer due to the very low oxidation energy of Fe(CN)_6_^4–^ (−0.19 eV, Figure S2). In other words, while hot holes at lower energies (≲−2 eV) have significantly larger fluxes to surfaces, their low injection probability means they have a limited role in the photoelectrochemical reaction. We propose that higher energy holes are more strongly oxidizing and more likely to participate in this reaction due to a tunneling barrier that they must overcome in order to reach the physisorbed molecule on the surface.^43^ This indicates that mostly the d-band-generated high-energy holes strongly participate in the oxidation reaction in our system, in agreement with suggestions from previous studies on hot-hole-driven reactions.^11−13^ Therefore, it is expected that these higher energy holes are mostly collected ballistically; otherwise, they immediately lose their energy under scattering events and would have insufficient energy to drive the reaction. The probability of carrier injection in our studied metal/molecule system may be different for molecules involved in inner-sphere reactions because of the required charge injection for chemical bond dissociation. Figure 4e shows the predicted IQE spectra from *F*_0_(*E*,*ℏ*ω) for the holes that reach to the top and side surfaces and the obtained *P*_inj_(*E*) in Figure 4d together with the experimentally determined IQEs for 16 and 25 nm thick Au NDs heterostructures (see 33 nm thick NDs in Figure S16). Our transport model and fitting approach for injection probability reproduced our experimental data well, in particular, our best-performing device, the 16 nm ND heterostucture. Almost the same computed IQE trend was obtained for the scattered hot holes (Figure S16). An analogous analysis applied to the hot electrons is reported in Supporting Information S.7 and Figure S18.

Based on our analysis of the injection probability, we also calculate the maximum possible IQE for our system that we could achieve for two upper-bound assumptions. First, we assume that the illumination and chemical reaction happen on the same side (e.g., bottom surface). The maximum IQE, in this case, is 0.12% (Figure S17a), which is still close to the experimentally obtained maximum value of 0.094% in our system (Figure 4e). Second, we assume that all of the generated holes immediately reach the top surface and are collected before any scattering events without any transport losses. The maximum IQE, in this case, is 5% (Figure S17b), which is ∼50 times higher than our obtained value. This emphasizes that transport is one of the main limiting factors in the performance of hot-carrier devices, which should be taken into account in future designs.

In summary, we established a fully controlled system to experimentally quantify the IQE in ultrathin (14–33 nm) single-crystalline plasmonic Au nanoantenna array Schottky photodiode and photoanode devices that operate via the collection of hot electrons (Au/TiO_2_) and both hot electrons and holes (Fe(CN)_6_^4–^/Au/TiO_2_), respectively. All of the Au nanoantenna array heterostructures were designed and fabricated to have plasmon resonance in the intraband region, allowing us to uniquely disentangle plasmon absorption and interband excitation effects. Our experimental IQE data combined with *ab initio* modeling revealed the role of intra- and interband decay processes and carrier transport over the nanometer size of the antennas, proposing an injection probability estimation for hot holes collected at the metal/electrolyte interface. Comparing the measured IQE spectra for the two devices having very close thicknesses (14 and 16 nm) showed a different magnitude and trend in IQEs, with an abrupt drop for the photodiode device and a continuous increase for the photoanode device in the interband regime. The magnitude difference suggested that the efficiency of the photoanodes is indeed controlled and limited by hot-hole collection at the metal/electrolyte interface. Our injection probability model indicated that mostly the d-band-generated high-energy holes participated in the oxidation reaction in our photoanode system and, particularly, are collected from the top {111} surface. Our transport model and fitting approach for injection probability showed that these hot holes and electrons are mostly collected ballistically at both the Fe(CN)_6_^4–^/Au and Au/TiO_2_ interfaces in our device with the highest collection efficiency for the thinnest nanoantenna photoanode device (16 nm) as compared to the thicker counterparts (25 and 33 nm). Our results and combined approach could reveal mechanistic insights into the generation, transport, and injection of hot carriers in hot-carrier-driven photocatalytic systems and be a guideline for the design of efficient devices operating in the ballistic regime, e.g., plasmon-driven artificial photosynthetic systems or optoelectronics.

## Supporting Information Available

Methods section, additional information about characterization of the redox molecule/Au/TiO_2_ system, characterization of the Au nanoantenna heterostructures, SECM approach for probing the photothermal heating at Au ND heterostructures, modeling the temperature increase in the nanostructure system, modeling the tip current response in photo-SECM, and a detailed analysis of IQE, transport, and injection probability results (PDF)
