# Supplementary material for: Transport and Interfacial Injection of d-Band Hot Holes Control Plasmonic Chemistry
Source: ACS Energy Lett. 2023 Sep 19;8(10):4242–50. doi: 10.1021/acsenergylett.3c01505 (PMC10580318; doi:10.1021/acsenergylett.3c01505)
Supplement: Supplementary file 1 — nz3c01505_si_001.pdf [file nz3c01505_si_001.pdf]

Supporting Information

Transport and Interfacial Injection of d-band Hot Holes Controls Plasmonic Chemistry

*Fatemeh Kiani[1], Alan R.Bowman[1], Milad Sabzehparvar[1], Can O.Karaman[1], Ravishankar Sundararaman[2], Giulia Tagliabue[1]**

[1] Laboratory of Nanoscience for Energy Technologies (LNET), STI, École Polytechnique Fédérale de Lausanne, 1015 Lausanne, Switzerland

[2] Department of Materials Science & Engineering, Rensselaer Polytechnic Institute, 110 8th Street, Troy, New York 12180, USA

**E-mail: giulia.tagliabue@epfl.ch*

23 pages, 18 figures

Contents

Supplementary Information 1. Methods

S1.1. Synthesis and device fabrication

S1.2. Optical measurements

S1.3. Photoelectrochemical and photocurrent measurements

S1.4. Numerical simulation

S1.5. Hot carrier transport and injection predictions

Supplementary Information 2. Characterization of the Redox molecule/Au/TiO_2 system

Supplementary Information 3. Characterization of the Au nanoantenna heterostructures

Supplementary Information 4. Scanning electrochemical microscopy (SECM) approach for probing the photothermal heating at Au NDs heterostructures

Supplementary Information 5: Modeling the temperature increase in the nanostructure system

Supplementary Information 6. Modeling the tip current response in photo-SECM

Supplementary Information 7. Detailed analysis of IQE – transport and injection probability

References

Supplementary Information 1: Methods

S1.1. Synthesis and device fabrication. In order to make a transparent semiconducting substrate, first a 50 nm-thick ITO film was deposited on a cleaned 1.5x1.5 cm^2, 520 µm-thick fused silica substrate in a sputtering system (Pfeiffer SPIDER 600). Subsequently, a 40 nm TiO_2 film was deposited onto the ITO surface with Alliance-Concept DP 650 sputtering instrument. The deposited TiO_2/ITO film was then annealed in air at 450 °C for 2 hours. Large area SC Au MFs were directly grown on borosilicate glass substrates by a halide and gap-assisted polyol process.[1] A PMMA wet-transferring method[2] was used for transferring the Au MFs onto the TiO_2/ITO-coated glass substrate. The sample was then exposed to an oxygen plasma (4 minutes, 500 W) to remove any PMMA residue left from the transferring step. Next, a layer of PMMA 495k A4 was spin-coated on the sample (120 nm) and backed for 5 minutes at 180 °C. Then, to fabricate the photoanode device, electron-beam lithography (EBPG5000ES system) was used to write the nanodisk array pattern on the Au MFs (100 pA beam current with exposure of 425 µC/cm^2) following by PMMA development (sequentially immersing in MIBK and IPA solutions for 1 min each). It is to mention that due to the transparent sample, two 5nm/100nm-thick Ti-Pt stripes were deposited at two sides of the substrate as the reflective layer for providing a correct height measurement to have the electron beam focused on the substrate surface during the writing. Next, ion beam etching (Veeco Nexus IBE350) was used to etch the Au flake area around the nanodisks (ultra low IBE process, -10° angle). The Remained PMMA on top of the nanodisks was removed by immersing the sample in pure acetone (20 min) and isopropanol (3 min) followed by rinsing with DI water and drying with N_2. Lastly, the sample was exposed to a mild oxygen plasma (30 s, 350 W) for further cleaning the chemical residues. To fabricate the photodiode device, focused ion beam (FIB) milling (30 kV Ga^+ beam) was used to make an 30x30 µm^2 Au stripe array from a transferred SC Au MF onto a TiO_2/ITO coated glass substrate. The FIB milling was performed with a FIB/SEM dual-beam instrument (Zeiss CrossBeam 540). A narrow film of Ti/Au (5 nm/100 nm) Ohmic contact was then deposited on the substrate surface close to the fabricated Au stripe array structure with a sputtering system (Alliance-Concept DP 650).

S1.2. Optical measurements. To record reflectance and transmittance spectra of the structures, an inverted microscope (Nikon Eclipse Ti2) was used in combination with a grating spectrometer (Princeton Instruments Spectra Pro HRS-500) equipped with a Peltier-cooled 2D CCD detector (Princeton Instruments PIXIS 256). A fiber-coupled broadband laser-driven white light source (Energetiq LDLS™) focused on the back focal plane (BFP) of a long working distance, high-NA objective (Nikon 60x, NA=0.7) to illuminate the sample from the bottom with a collimated light. The reflected light was directed to the spectrometer. The

measured reflectance was normalized to the reflectance of a silver mirror (ThorLabs, PF10-03-P01) and multiplied by the known reflectance of the mirror. Also, the background was subtracted from all the measurements. The transmittance measurements were carried out by top illumination of the sample through a bright field condenser lens. The sample was faced down for the transmission and faced up for the reflection measurements, ensuring the beam first hit the substrate and then Au nanostructures to implement the practical illumination condition required for the liquid-state and solid-state experiments. The reverse direction was applied for the front illumination condition.

S1.3. Photoelectrochemical and photocurrent measurements. To perform liquid-state photoelectrochemical measurements, a custom-built photo-SECM set-up is integrated by adding to an inverted optical microscope (Nikon Eclipse Ti2), a home-built electrochemical reaction cell, a bi-potentiostat (Biologic SP-300), and an ultramicroelectrode (UME) tip connected to a micro/piezo scanner assembly (MMP1/Nano-F450, Mad City Labs). Pt UME tips were fabricated by heat-sealing and hard pulling of a Pt micro-wire (25 µm, Goodfellow) within a borosilicate glass capillary (1mm ID, 0.5mm OD, Sutter Instruments) with a laser puller machine (P-200, Sutter Instruments), followed by physical contacting of a Cu wire to the Pt wire using a silver epoxy. The laser-pulled UMEs were perfected on an ultrafine polishing plate (BV-10, Sutter Instruments) under video monitoring. A Pt wire (0.5 mm diameter) and a leak-free Ag/AgCl electrode (LF-1, 1 mm OD, Innovation Instrument) were used as counter and reference electrodes, respectively. Potassium hexacyanoferrate (II) trihydrate ($K_4Fe(CN)_6.3H_2O$, 99.95%), Potassium hexacyanoferrate (II) ($K_3Fe(CN)_6$, 99.98%) ,and potassium chloride (KCl, 99%) were purchased from Sigma-Aldrich and used as received. The optimized concentration of 4mM $Fe(CN)_6^{4-}$/0.25M KCl was found for the photo-SECM experiments based on the stability of the Au NDs and the Pt UME tip response. The tip current gets time-dependent with no diffusion-limiting regime (see **Figure S2.c**) and thus a large transient time and hysteresis at a higher concentration of 10mM as compared to 4mM concentration (see **Figure S2.b**). A high power supercontinuum white light laser (NKT Photonics) was used for plasmon excitation. A tunable wavelength filter (SuperK VARIA) was employed to modulate the excitation wavelength and power in a wide spectral range of 450 to 840 nm with the bandwidth of 20 nm. An optical shutter (SH1, Thorlabs) was used to chop the incident light. A combination of two lenses was added to the optical path to provide a bottom illumination on the sample with a 30 µm diameter collimated beam. To define the beam diameter, first a CCD image of the laser spot was recorded and then fitted by a two-dimensional Gaussian. Spot diameter here is as the intensity that falls to $\frac{1}{e^2}$ of the maximum intensity. The solid-state photocurrent measurements were carried out by utilizing piezoelectric microprobes (Imina

Technologies, miBots™) to electrically connect the sample and record the short-circuit current. Note that a polarizer (WP25M-VIS,Thorlabs) was added to the optical path to provide a polarized beam perpendicular to the Au stripes. The current-voltage (*I-V*) and time-trace of the photocurrent (*I-t*) curves were recorded through a Keithley 2450 SourceMeter.

S1.4. Numerical simulation. The electromagnetic simulations were performed using the RF module of the COMSOL Multiphysics v5.6 to simulate absorption spectra as well as 3D internal electric field distributions across the volume of the nanoantennas. The latter was used as an input in the subsequent hot carrier transport calculation code. A 3D unit cell model, consisting of one Au nanoantenna (disk or stripe geometry) on TiO_2/ITO/fused silica substrate surrounded with a top layer of water (for disk) or air (for stripe), was simulated by setting the unit cell width equal to the array periodicity (200 nm for disks and 230 nm for stripes) and unit cell length equal to 200 nm and 300 nm for disks and stripes, respectively. Perfect magnetic conductor and perfect electric conductor boundary conditions were used at the sidewalls of the unit cell. A port boundary condition with the excitation "ON" was set at the bottom of the unit cell for the back illumination with a normal incident plane wave (450-850 nm) with electric field polarization perpendicular to the stripe length axis as well as for recording the reflected wave. A second port boundary condition without excitation was used at the top of the unit cell to record the transmitted wave. The absorbed power was calculated by volume integration of the electromagnetic power loss density over the nanoantenna volume. The wavelength-dependent complex refractive indices for SC Au, TiO_2 and ITO were taken from refs [3], [4], and [5]. A 2D diffusion COMSOL model was also implemented to simulate the tip current (I_{Tip}) response in photo-SECM experiments. Simulation details are provided in the **Supplementary information 6**.

S1.5. Hot carrier transport and injection predictions. Starting from the electric field distribution from the electromagnetic simulations, we predict the initial spatially-resolved energy distribution of hot carriers and their subsequent transport accounting for electron-electron and electron-phonon scattering using the Non-Equlibrium Scattering in Energy and Space (NESSE) simulation framework.[6] Briefly, this technique uses *ab initio* simulations of the optical excitation due to direct and phonon-assisted transitions to predict the initial carrier distribution, and then evolves spatially-resolved carrier energy distribution using the Boltzmann transport equation with a collision integral parameterized to first-principles electron-phonon and electron-electron scattering. The NESSE formulation predicts the carrier flux incident on the surfaces of the structure before scattering, after scattering once, twice, etc., allowing us to separate the

contributions due to scattered and unscattered carriers. See Ref. [6] for a detailed description of the algorithm and the underlying first principles calculations used to parameterize the carrier generation and transport parameters.

In the manuscript the IQE is measured for a range of situations and the fluxes at the surfaces are calculated as $F_i(E, \hbar\omega)$, where subscript i corresponds to situation i (denoting excitation wavelength and nanostructure excited). We assume that the probability of a hole transferring from the nanostructure and causing a chemical reaction, $P_{inj}(E)$, is only a function of the hole's energy and not of the excitation wavelength or nanostructure shape (noting we assume transfer occurs at the exposed top {111} and side {110} surfaces). In this case we can state that

$$IQE_1(\hbar\omega) = \int (F_{top_1}(E,\hbar\omega)P_{inj,top}(E) + F_{sid\ ,1}(E,\hbar\omega)P_{inj,side}(E))dE \qquad \text{(S1)}$$

$$IQE_2(\hbar\omega) = \int (F_{top_2}(E,\hbar\omega)P_{inj,top}(E) + F_{side,2}(E,\hbar\omega)P_{inj,side}(E))dE \qquad \text{(S2)}$$

$$\vdots$$

for all i situations, where the integral is over all hole energies and top and side refer to the fluxes and injection probabilities at the top and side.

We adopted a stochastic fitting approach to find the form of $P_{inj}(E)$ which best reproduced the IQEs. Specifically, we split the hole energy space into M distinct, equally spaced points (e.g. $E_{minimum}$,E_{mean}, $E_{maximum}$ for M=3). At each of these points we randomly assigned a value to P_{inj} between 0 and 1, and between these points we carried out a linear interpolation to find P_{inj} at all energies. We always treated top and side injection probabilities separately. We calculated the difference between computed and measured IQEs. We then randomly selected one of the defined points in energy space and varied this point until the best possible agreement between experiment and theory was found (within a tolerance). We repeated this approach, varying different defined points, until the difference between computed and measured IQEs was no longer changing. This gave us a set of P_{inj} values which reproduced experimental data well. Finally, we repeated this fitting process 200 times, to obtain a spread in the P_{inj} values which reproduce the experimental data well. In the main text we present the average and standard deviation of P_{inj} values.

To carry out this fitting approach it is necessary to select a value for M, the number of distinct points to fit. Here we selected some M, carried out the above algorithm, and then calculating the average difference between calculated and measured IQEs. We then increased M until the difference between calculated

and measured IQEs was not significantly changing (< 5 %) when M was further increased. In our simulations we found M=7 reproduced the experimental data well.

In order to calculate the maximum IQE if the photon absorption and chemical reaction were to occur on the same surface, we used our extracted values of P_{inj} to calculate the quantity

$$IQE_{max,i}(\hbar\omega) = \int (F_{bottom,i}(E,\hbar\omega)P_{inj,bottom}(E) + F_{side,i}(E,\hbar\omega)P_{inj,side}(E))dE \quad \text{(S3)}$$

where $F_{bottom,i}$ is the calculated hole flux at the bottom surface i.e. the side where holes were generated. The $IQE_{max,i}$ values are plotted in **Figure S17.a**.

In order to calculate the maximum IQE that could ever be obtained from this type of system we calculated

$$IQE_{limit}(\hbar\omega) = \int P_{gen}(\hbar\omega,E)P_{inj,top}(E)dE \quad \text{(S4)}$$

where $P_{gen}(\hbar\omega,E)$ is the (calculated) number of holes generated at each hole energy E as a function of the excitation wavelength, $\hbar\omega$, per absorbed photon. $P_{gen}(\hbar\omega,E)$ is taken from reference[7]. This is plotted in **Figure S17.b**.

Supplementary Information 2: Characterization of the Redox molecule/Au/TiO_2 system

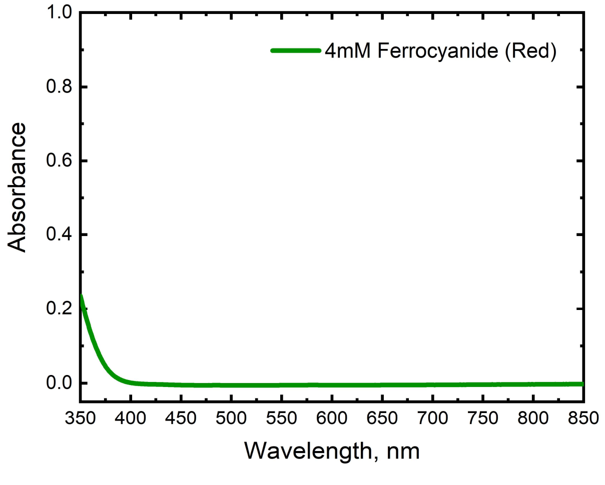

Figure S1. UV-Vis spectrum of 4mM $K_4Fe(CN)_6$ in 0.25M KCl aqueous solution. Ferrocyanide molecule does not absorb visible-light.

We measured the Fermi level (E_F) of our $Fe(CN)_6^{4-}$/Au/TiO_2 system and the *HOMO* level of the $Fe(CN)_6^{4-}$ molecule by open-circuit potential and cyclic voltammetry measurements in the electrolyte media. A value of ~ - 0.0012 V vs Ag/AgCl (-4.64 eV vs vacuum), and 0.187 V vs Ag/AgCl (-4.83 eV vs vacuum) was measured for the E_F and *HOMO* levels, respectively (**Figure S2**).

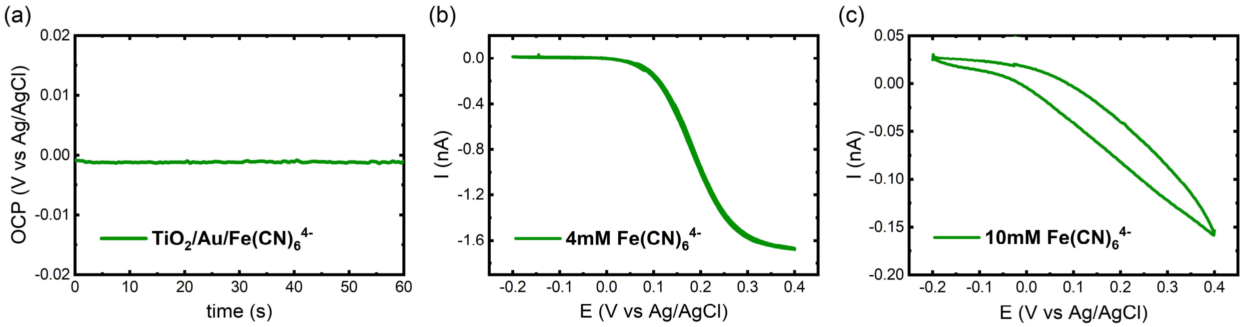

Figure S2. (a) Open-circuit potential (OCP) plot showing the E_{OCP} of Au/TiO_2 working electrode in the presence of 4mM $Fe(CN)_6^{4-}$ after 30 min equilibrium time delay. (b) Steady-state cyclic voltammogram of a 1.9 μm UME tip showing the HOMO level of $Fe(CN)_6^{4-}$ that can be estimated from the half-wave potential. (c) Cyclic voltammogram of a 1.9 μm UME tip in 10mM $Fe(CN)_6^{4-}$ concentration electrolyte.

Supplementary Information 3: Characterization of the Au nanoantenna heterostructures

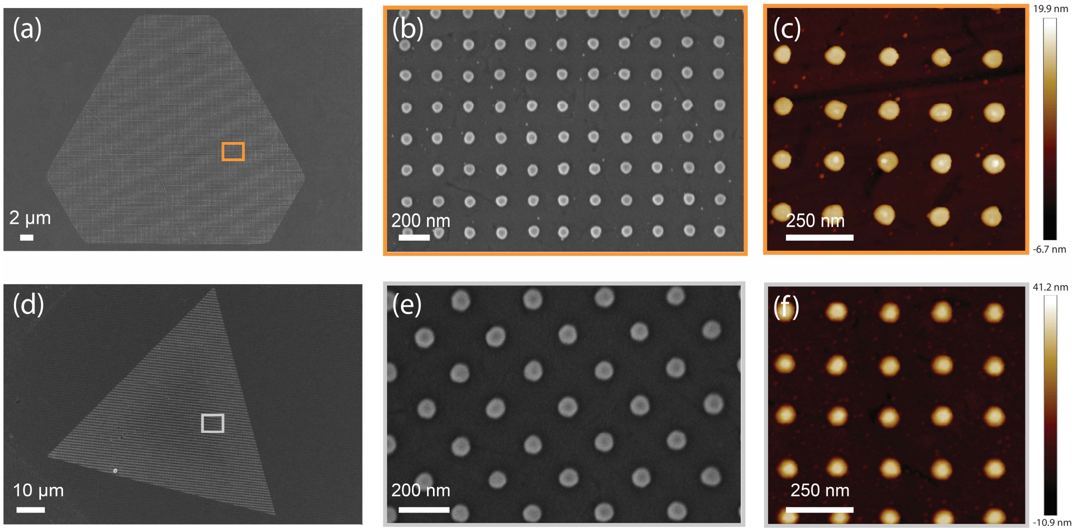

Figure S3. SEM and AFM images of the fabricated Au nanodisks (NDs) arrays from Au micro-flakes with thicknesses of 16 nm (a, b, and c) and 33 nm (d, e, and f) on a TiO_2/ITO-coated fused silica substrate.

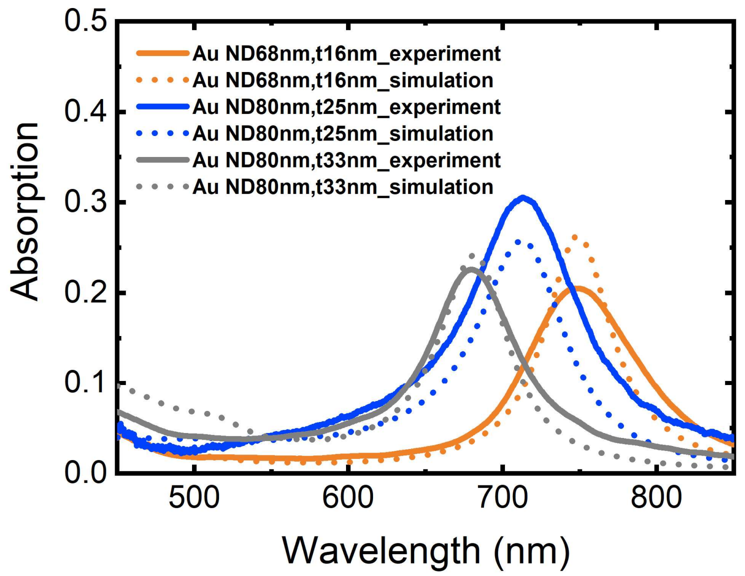

Figure S4. Measured (solid lines) and simulated (dotted lines) absorption spectra in front illumination condition for Au NDs having diameters of 68 and 80 nm and thicknesses of 16, 25 and 33 nm, respectively.

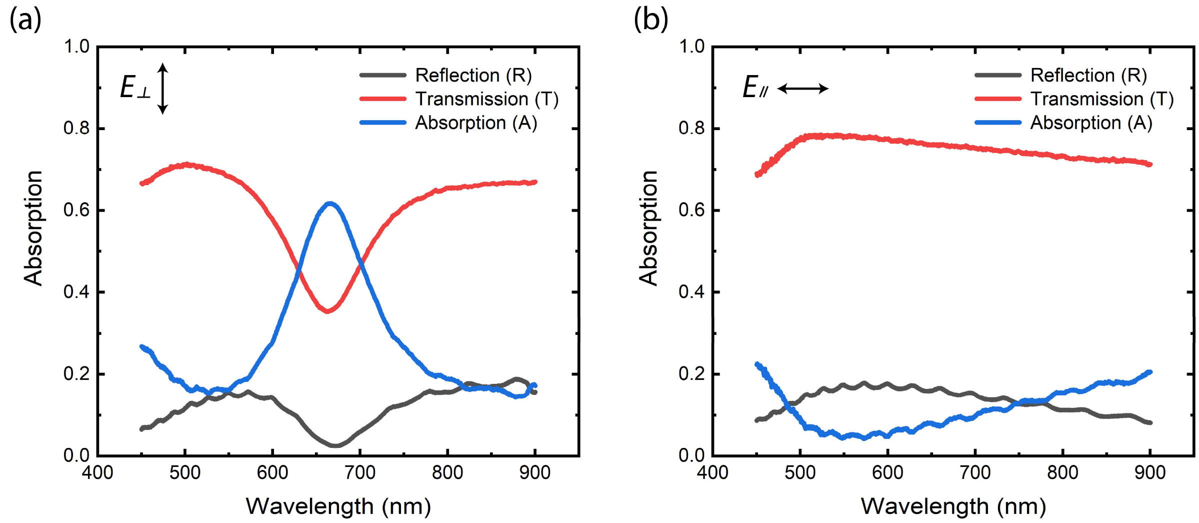

Figure S5. Reflection (R) and transmission (T) spectra of the 14 nm-thick Au stripe measured with a polarized light (a) perpendicular and (b) parallel to the stripes. The blue curves show the absorption spectrum determined as A= 1- T- R.

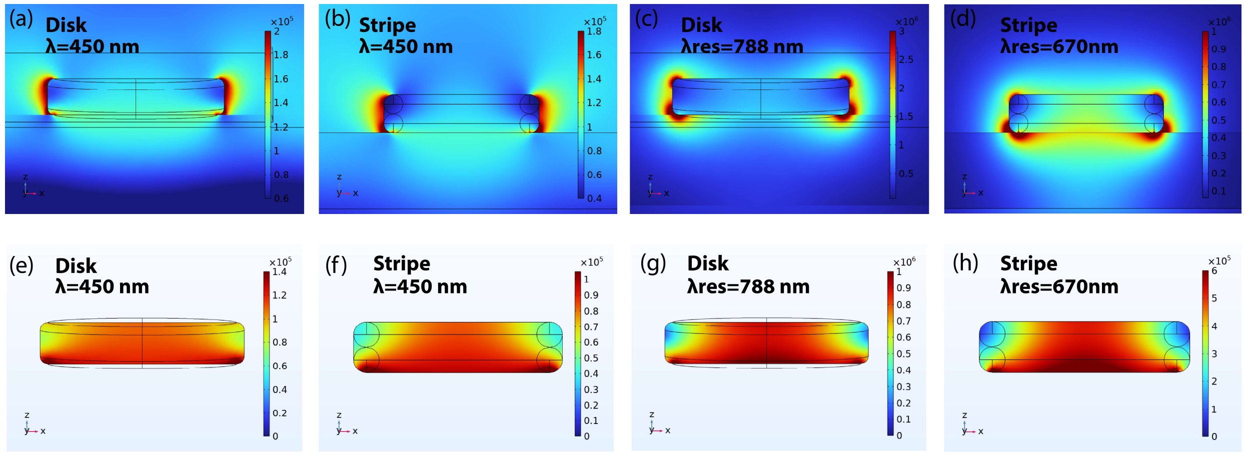

Figure S6. Simulated Electric field norm profile for (a-d) overall (inside and outside) and (e-h) inside of the disk (diameter=68nm, thickness=16nm) and stripe (width=70nm, thickness=14nm) geometry at on-resonance and off-resonance illumination conditions. The electric field profile is identical at the bottom interface for both systems.

Supplementary Information 4: Scanning electrochemical microscopy (SECM) approach for probing the photothermal heating at Au NDs heterostructures

To determine whether hot carriers or photothermal heating effects enhance the photo-oxidation reaction at the Au NDs surface in our experiments, we performed SECM measurements in competition and substrate generation/tip collection (SG/TC) modes in an electrolyte solution containing 4mM $Fe(CN)_6^{3-}$ (oxidant, *Ox*) and $Fe(CN)_6^{4-}$ (reductant, *Red*) redox molecules and 0.25 M KCl. As a photo-oxidation reaction happens at the substrate, the tip was biased at an oxidizing potential ($E_T >> E_0$) of 0.4 V vs Ag/AgCl for the competition and at a highly reducing potential ($E_T << E_0$) of 0 V vs Ag/AgCl for the SG/TC experiments. The substrate was at open-circuit condition, grounded, and illuminated from the bottom at a broad range of illumination intensities from 5 to 630 W/cm^2 at the resonance wavelength of the Au NDs structure. The time trace of i_{Tip} for SG/TC and competition experiments are plotted in **Figure S7.a** and **b**, respectively. By increasing the power intensity, the light-induced change in i_{Tip} increases in the SG/TC mode because more oxidant species are supplied to the tip electrode reaction under illumination while it decreases in the competition mode because less reductant species are supplied to the tip electrode reaction. In fact, the steady state, mass transport–limited anodic tip current ($i_{a,\infty}$) and cathodic tip current ($i_{c,\infty}$) in **equations S5** and **S6** are a function of both the mass transport coefficient and local concentration of reactants,

$$i_{a,\infty} = FAm_{Red}C^*_{Red} \quad \text{(S5)}$$

$$i_{c,\infty} = FAm_{Ox}C^*_{Ox} \quad \text{(S6)}$$

where F is the Faraday constant, A is the area of the tip electrode, m_{Ox} and m_{Red} are the mass transport coefficient of oxidant and reductant, respectively and C^*_{Ox} and C^*_{Red} are the bulk concentration of *Ox* and *Red*, respectively. This means that at open-circuit condition, the observed light-induced change in i_{Tip} in **Figures S6a,b** can be due to both the increase in mass transport rate of the species due to the photothermal heating and the change in concentration of the species because of the hot hole-driven photo-oxidation reaction at the substrate. To isolate these two effects, an alternative approach is to determine the ratio of the local concentration of *Ox* and *Red* using **equations S5** and **S6**.[8,9]

$$\frac{C_{Ox}}{C_{Red}} = \frac{m_{Red}}{m_{Ox}}\frac{i_{c,\infty}}{i_{a,\infty}} \quad \text{(S7)}$$

Here the ratio of the mass transport of the two species remains constant ($m_{Red}/m_{Ox} = 0.92$) as a function of illumination intensity, mass transport rates of both oxidant and reductant are equally affected by temperature increase.[8,9] Therefore, comparing the magnitude of $i_{c,\infty}$ and $i_{a,\infty}$ when the substrate is at open circuit condition, enables us to deconvolute the hot hole-driven photo-oxidation from the interference of photothermal heating effect on mass transport of the species. The C_{Ox}/C_{Red} at different illumination intensities are calculated using **equation S7** and plotted in **Figure S7.c**. We note that photothermal heating of the substrate not only affects the mass transport rate of the species but can also shift the standard potential of the redox molecule (E^0). It has been shown that the E^0 of the $Fe(CN)_6^{3-/4-}$ linearly decreases as the temperature increases.[8,9] The relationship between C_{Ox}/C_{Red} and the potential shift (ΔE) can be evaluated based on the Nernst equation (**eq. S8**).

$$\Delta E = E^0 - E_s = -\frac{RT}{F} ln\frac{C_{Ox}}{C_{Red}} \quad \text{(S8)}$$

where E_s is the substrate potential, T is the temperature, R is the Boltzmann constant and F is the Faraday constant. If the local heating exists in the system, ΔE should decrease linearly with temperature, and thus with excitation intensity because temperature scales linearly with illumination intensity. As a result, an exponential dependence is expected for C_{Ox}/C_{Red} with excitation intensity, as was reported before for thermally-enhanced photo-oxidation reactions.[8,9] In our case, we observed a linear dependency with the excitation intensity up to 330 W/cm^2, suggesting that plasmon-excited hot holes generated at Au NDs surface drive the photo-oxidation reaction within this power intensity range. Upon increasing the power intensity to 630 W/cm^2, the C_{Ox}/C_{Red} ratio starts to deviate from this linearity with fluctuations between

409 and 630 W/cm^2. This observation is consistent with our cyclic voltammetry (CV) measurement results in **Figure S7.d**, where E^0 (determined from half-wave potential, $E_{1/2}$) remains unchanged up to 330 W/cm^2 but shifts by approximately 38 mV when the intensity is increased to 630 W/cm^2. We note that the maximum power intensity used for determining IQEs for different heterostructures from our competition-mode photo-SECM experiments (**Figure 2** in the manuscript) was 14 W/cm^2 which is expected to not have photothermal effects as we are in the linear range of the c_O/c_R vs power intensity (indicated by the green area in **panel (c)**).

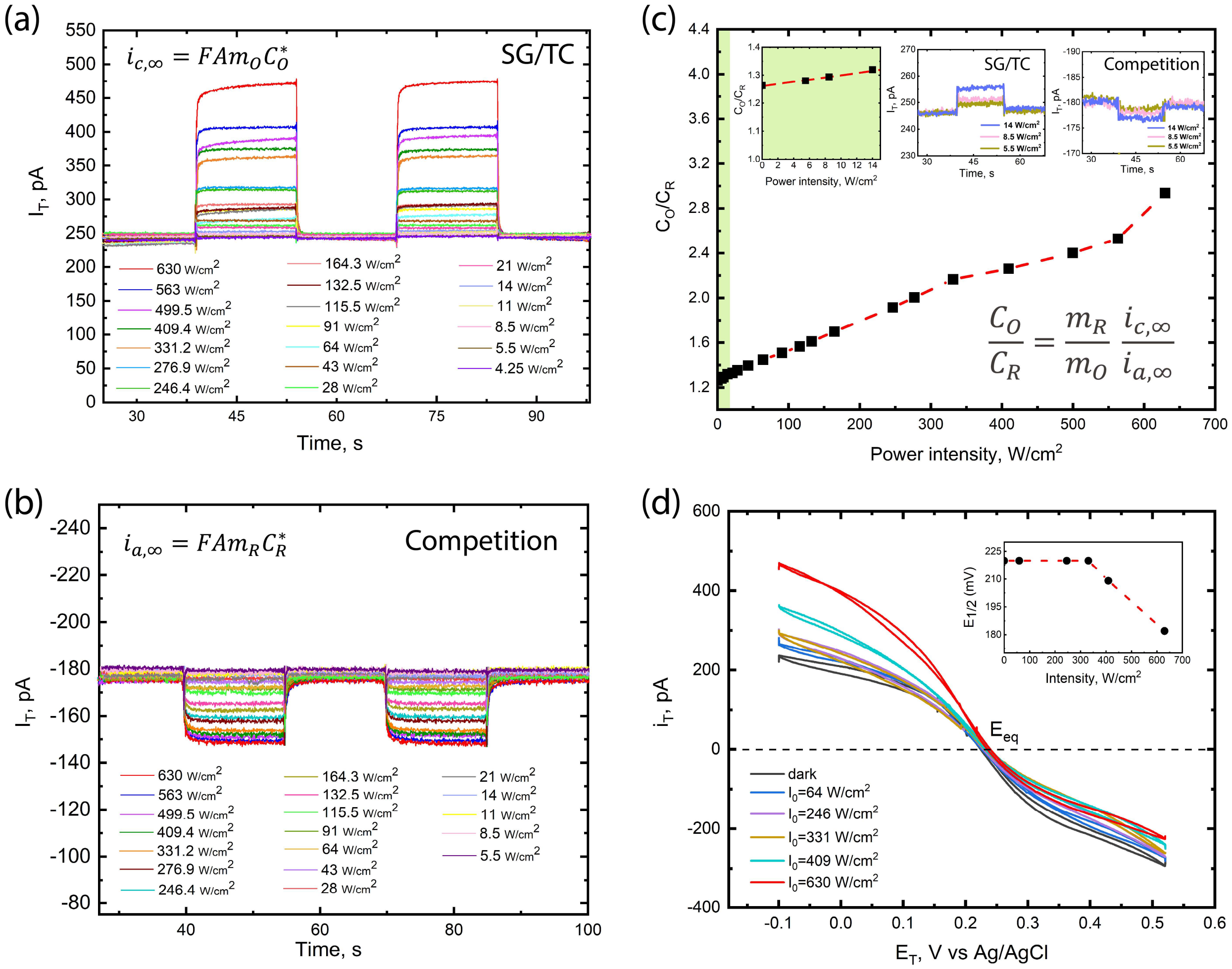

Figure S7. Photo-SECM results for probing photothermal heating at Au NDs with a diameter of 80 nm and thickness of 25 nm. Time traces of tip current (i_{Tip}) in (a) SG/TC (E_T= 0 V vs Ag/AgCl) and (b) competition (E_T= 0.4 V vs Ag/AgCl) modes of operation at different power intensities. (c) Oxidant to reductant concentration ratio (C_O/C_R) vs. power intensity relationship. The green shaded area and the inset represent the power intensities used for photo-SECM experiments for IQE analysis in the main text. (d) Cyclic-voltammograms obtained in dark and under illumination condition at different power intensities.

The inset in (d) shows the half-wave potential ($E_{1/2}$) plot vs power intensity. All the experiments are performed with a 1.2 µm Pt UME tip at a tip-to-substrate distance of 5 µm, and a beam spot size of 30 µm (λ = 746 nm) in an aqueous electrolyte solution containing 4mM $Fe(CN)_6^{3-}$ (Ox) and $Fe(CN)_6^{4-}$ (Red) redox molecules and 0.25 M KCl. The substrate is at open circuit and grounded.

Supplementary Information 5: Modeling the temperature increase in the nanostructure system

Here we present three arguments considering the heating of nanoparticles to show it remains insignificant in our experiments.

1. **Maximum heating from a single laser pulse**

Here we consider the limiting case that all energy from a laser pulse goes into heating the sample. We recorded the intensity of the laser pulse, I, and here we note that in our measurements the laser frequency, $f = 80$ MHz. We can therefore say that the energy per pulse, per unit area is

$$E_{pulse} = \frac{I}{f} \tag{S9}$$

We can equate the power absorbed in a single nanodisk with the temperature increase this will cause, that is:

$$aE_{pulse}d^2 = VC\Delta T \tag{S10}$$

where a is the absorption of the gold, d is the distance between two disks, V the volume of a disk, C the heat capacity of gold (per unit volume) and ΔT the temperature. We can also state that $V = \pi r^2 t$, where ρ is the density of gold, r the disk radius and t the disk thickness. Re-arranging we find that

$$\Delta T = \frac{Iad^2}{f\pi r^2 tC} \tag{S11}$$

We calculate ΔT caused by our experiments at the strongest sample absorption (see **Figure S8** for values), we obtain the (maximum) temperature increase due to excitation by a single pulse of 0.22 K. We have

chosen the parameters (wavelength, disk size, absorption) that gives the maximum temperature increase of any of the nanostructures measured for the IQE analysis in the manuscript.

2. Time evolution of nanoparticle temperature due to heating from a single pulse

The metal can be treated as two coupled thermal systems made up of the electron and the phonon baths when nonequilibrium electron heating is being considered[10,11]. A phenomenological description of the electron gas can be produced by dividing the electron distribution into a high-density thermalized distribution and a very-low-density nonthermalized distribution due to the extremely small electron density that was first perturbed by the pump pulse. This nonthermalized part acts as a heat reservoir and decays by exchanging energy with the Fermi component until it reaches thermal equilibrium. Electron - phonon scattering is then used to transfer energy to the lattice. Then, by taking into the energy content of the electron gas, the evolution of the coupled electron-phonon system can be described by the following three coupled differential equations:

$$\frac{\partial N(t)}{\partial t} = -aN(t) - bN(t) + P_{abs}(t) \tag{S12}$$

$$C_e \frac{\partial T_e}{\partial t} = -G\big(T_e(t) - T_l(t)\big) + aN(t) \tag{S13}$$

$$C_l \frac{\partial T_l(t)}{\partial t} = G\big(T_e(t) - T_l(t)\big) + bN(t) \tag{S14}$$

where C_e and C_l are the electron and lattice heat capacities, T_e and Tl are the electron and lattice temperatures, G is the electron-phonon coupling constant, N is the variable describes the energy density stored in the nonthermalized part of the distribution, a is the average scattering rate of the nonthermalized electrons, and b is the electron-phonon coupling rate. The constant values are taken from ref [10], [11] and [12]. P_{abs} is the pump-pulse power absorbed in the unit volume of the metal and it is expressed as a function of E_{pulse} by

$$P_{abs} = \sqrt{\frac{2}{\pi}} \frac{U_{abs}(\lambda)}{\tau_p} exp(-\frac{t^2}{\tau_p^2}) \tag{S15}$$

Where

$$U_{abs}(\lambda) = \frac{\frac{Abs(\lambda)E_{pulse}}{\pi r_{beam}^2} \frac{\pi r^2}{C}}{\pi r^2\ t} \tag{S16}$$

Where τ_p is the pulse length, $r_{beam\backslash}$ is the beam radius and C is the coverage factor defined as the ratio between the area of the ND and the unit cell. By simulating the experimental conditions, we observe the

maximum light induced ΔT_l in all set of experiments is around 0.2K while ΔT_e is around 10K which happens in 10 ps after the excitation as can be seen in **Figure S8.a**.

3. Steady state temperature increase reached due to laser

To account for the collective heating in the arrayed NDs[13] and heating due to all laser pulses (i.e. steady state conditions), we used the method described by Naef et al.[14] and simulated the experimental conditions (**Figure S8**). As can be seen in **Figure S8.b**, the temperature profile is the complex combination of the Gaussian shape of the laser pulse and the collective heating due to the arrayed NDs. The maximum ΔT_l is around 0.2 K which happens in the center of the array. Based on the results, the temperature change in our experiments is negligible and do not have any practical effect in our results.

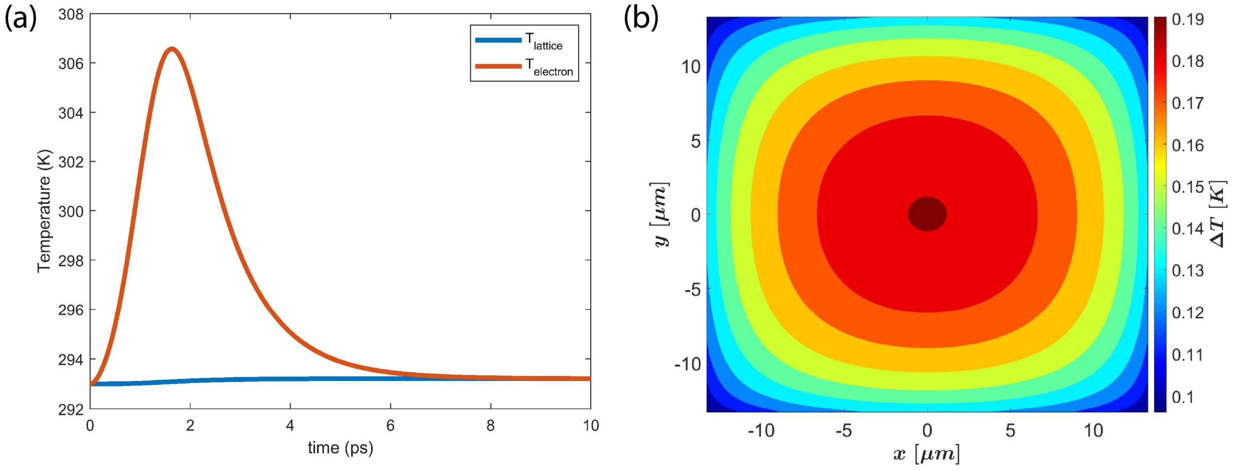

Figure S8. (a) Time dynamics of the T_e and T_l in Au NDs in the experimental conditions of Abs(780nm)=0.54, Power =100 µW, repetition rate= 80 MHz, pulse length=1ps, laser beam diameter=30 µm, number of NDs=17689, ND diameter=68 nm, ND thickness=16 nm, periodicity= 200 nm, (b) Simulation to compute the collective heating at equivalent thermal conductivity of the host medium= 1.95 W/mk.

Supplementary Information 6: Modeling the tip current response in photo-SECM

To model the tip current response (i_{Tip}), a 2D simulation was performed using COMSOL Multiphysics v5.6. **Figure S9** shows the 2D axisymmetric geometry of the simulation space and parameters defining the diffusion problem, representing the UME tip positioned above the illuminated area of our plasmonic structure. The steady-state diffusion of the redox species (i.e. $Fe(CN)_6^{3-/4-}$) in this model was simulated

using the transport of diluted species (tds) module and solving the differential equation relating to the Fick's second law of diffusion in cylindrical coordinate,

$$\frac{\partial^2 c_i}{\partial r^2} + \frac{1}{r}\frac{\partial c_i}{\partial r} + \frac{\partial^2 c_i}{\partial z^2} = 0; 0 \le r \le r_s,\ 0 \le z \le l \tag{S17}$$

where r and z are the coordinates directions, and r_s and l are the width and the height of the simulation space, respectively. c_i in this model is the concentration of reductant for a competition SECM mode with a diffusion-controlled oxidation reaction at the tip electrode and a photo-oxidation reaction with a k_{eff} conversion rate at the illuminated area of the substrate, and no flux at the insulating boundaries and non-illuminated areas. The considered boundary conditions are reported below:

$$\frac{c_R}{c_O} = 0,\ D_O\frac{\partial c_O}{\partial n} = -D_R\frac{\partial c_R}{\partial n}; 0 \le r \le a; z = d; (tip\ electrode) \tag{S18}$$

$$c_R = c_{bulk},\ c_O = 0; r_g < r \le r_s,\ z = l; r = r_s,\ 0 \le z \le l; (bulk) \tag{S19}$$

$$\frac{\partial c_i}{\partial n} = 0; a < r \le r_g,\ z = d; r = r_g,\ d \le z < l; w_0 < r < r_s,\ z = 0; (insulator) \tag{S20}$$

$$D_O\frac{\partial c_O}{\partial n} = -D_R\frac{\partial c_R}{\partial n} = k(r)c_R; 0 < r \le w_0,\ z = 0; (substrate\ electrode) \tag{S21}$$

$$k(r) = k_{eff}(e^{-\frac{2r^2}{w_0^2}}) \tag{S22}$$

where D_O and D_R are the diffusivity of ferri- and ferrocyanide, respectively.[15] *a* is the radius of the Pt core of UME tip, *d* is the tip-substrate distance, c_{bulk} is the bulk concentration of reductant, r_g is the radius of the glass sheath of the UME tip, $\partial c/\partial n$ is the normal derivative of concentration, w_0 is the radius of the beam spot, $k(r)$ depicts the Gaussian profile of the rate constant within the laser beam spot, and k_{eff} is the effective rate constant of the substrate at the beam center. k_{eff} was set equal to 0 cm/s under dark condition. A high reaction rate constant was defined at the tip electrode ($k_{tip} = 100\ m/s$) to ensure a diffusion-limited condition for the competition mode. The k_{eff} of the light-driven photochemical reaction at the substrate (**eq. S23**) was swept to calculate the concentration profile and diffusive flux of the redox molecules to the tip. Then, the i_{Tip} and the $i_{sub,photo}$ were calculated by integrating the normal diffusive flux of the *Red* to the tip electrode (**eq. S24**) and to the illuminated substrate (**eq. S25**), respectively.

$$Fe(CN)_6^{4-}\ (Red) - e^- \rightarrow\ Fe(CN)_6^{3-}\ (Ox) \tag{S23}$$

$$i_{Tip} = 2\pi FDR\int_0^a \left[\frac{\partial c_R}{\partial n}\right]_{z=d} rdr \tag{S24}$$

$$i_{sub,photo} = 2\pi FDR \int_0^{w_0} \left[\frac{\partial c_R}{\partial n}\right]_{z=0} rdr \qquad \text{(S25)}$$

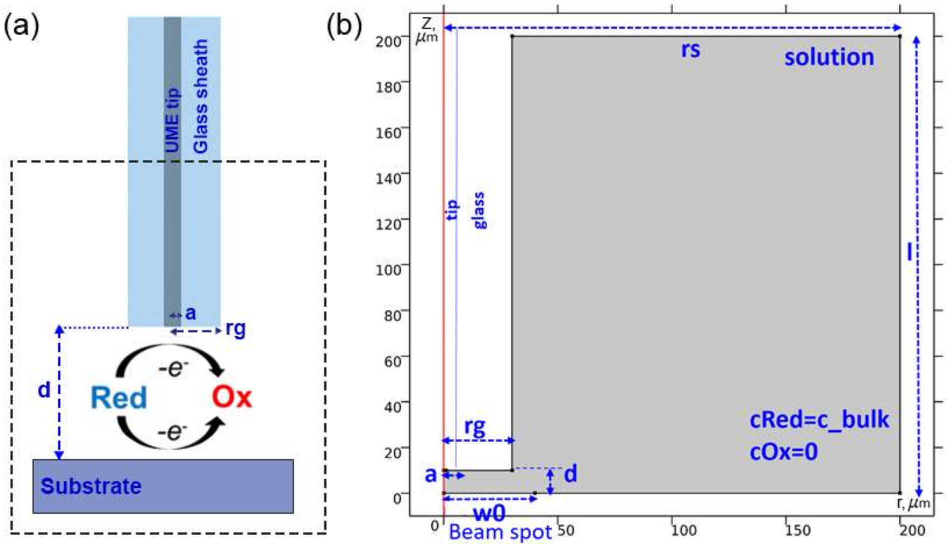

Figure S9. Modeling the tip current response in photo-SECM. (a) Schematic of the SECM experiment in competition mode. The black dashed counter depicts the simulation domain. (b) Geometry of the simulation space and parameters defining the diffusion problem: a is the tip radius, rg is the sheath radius, d is the tip-to-substrate gap distance, and w0 is the laser beam spot radius.

Figure S10.c shows the simulated calibration curves. The blue curve shows how the calculated $i_{Tip}/i_{Tip,dark}$ changes as a function of the k_{eff}. As expected, a lower $i_{Tip}/i_{Tip,dark}$ value corresponds to a higher k_{eff} for a competition SECM mode. Faster oxidation kinetics at the substrate leads to a lower concentration of *Red* available to react at the tip and thus a corresponding decrease in i_{Tip}.[16,17] The changing trend of the $i_{sub,photo}$ with k_{eff} is shown as the red curve in **Figure S10.c**. The $i_{sub,photo}$ becomes larger as the k_{eff} increases, and eventually reaches the diffusion-limited region. Using the simulated calibration curves, the experimentally measured i_{Tip} data can be converted to k_{eff} and $i_{sub,photo}$ under different illumination powers (**Figure S10.d**). The external quantum efficiency (EQE) of the photoelectrochemical reaction (**eq. S23**) is determined from the slope of the linear fit to the $i_{sub,photo}$ vs illumination power curves for each excitation wavelength using **equation S26**.

$$EQE = \frac{i_{sub,photo}/e}{P/h\nu} \qquad \text{(S26)}$$

where $i_{sub,pho}\ /P$ is the slope of the substrate photocurrent vs power plot, e is the charge of electron, and hv is the photon energy.

The internal quantum efficiency (IQE) and ΔIQE are determined using **equations S27** and **S28**, respectively.

$$IQE = \frac{EQE}{A} \tag{S27}$$

$$\Delta IQE = IQE\sqrt{(\frac{\Delta EQE}{EQE})^2 + (\frac{\Delta A}{A})^2} \tag{S28}$$

where A is absorption and Δ corresponds to the error in a quantity.

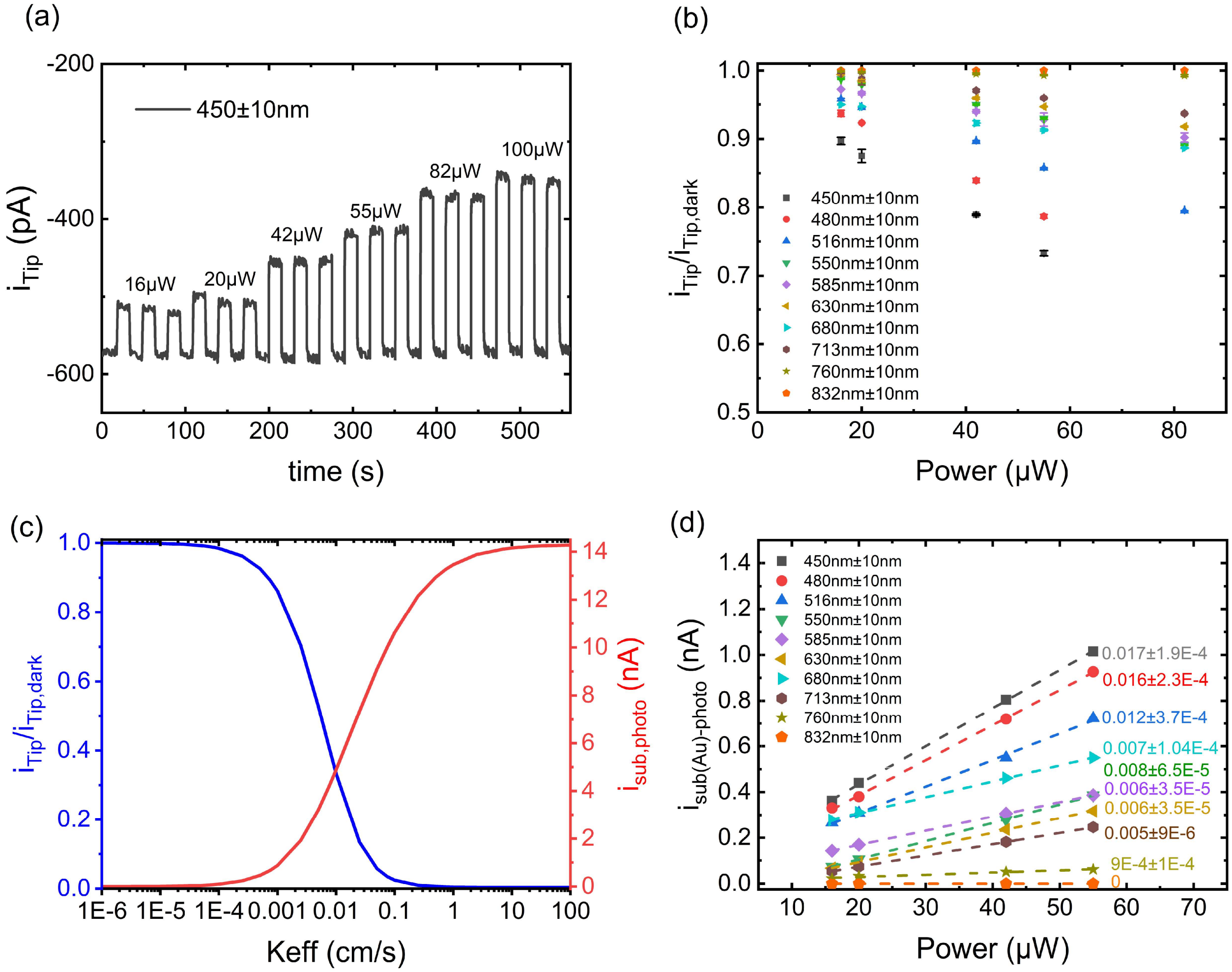

Figure S10. (a) Time trace of tip current (i_{Tip}) obtained from a 1.2 μm radius Pt UME upon illumination of the 25 nm-thick Au NDs array with the excitation wavelength of 450±10 nm up to 100 μW. The tip to substrate distance was 2.5 μm. (b) Measured $i_{Tip}/i_{Tip,dark}$ response as a function of power for a broad

excitation wavelength range of 450 to 832 nm. (c) Simulated calibration curves correlating $i_{Tip}/i_{Tip,dark}$, $i_{sub,photo}$, and K_{eff} obtained by the COMSOL diffusion model. (d) Extracted substrate photocurrent ($i_{sub,photo}$) vs power, using the calibration curves in (c) and measured data of $i_{Tip}/i_{Tip,dark}$ in (b) for each excitation wavelength. The slope of the linear fit (dashed lines) to these data is mentioned for each excitation wavelength.

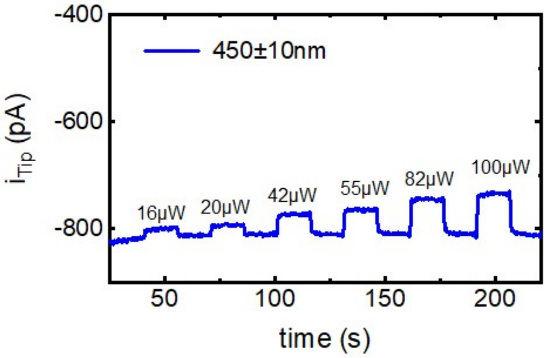

Figure S11. Measured i_{Tip} under illumination of TiO_2/ITO substrate with 450±10 nm excitation wavelength.

Supplementary Information 7: Detailed analysis of IQE – transport and injection probability

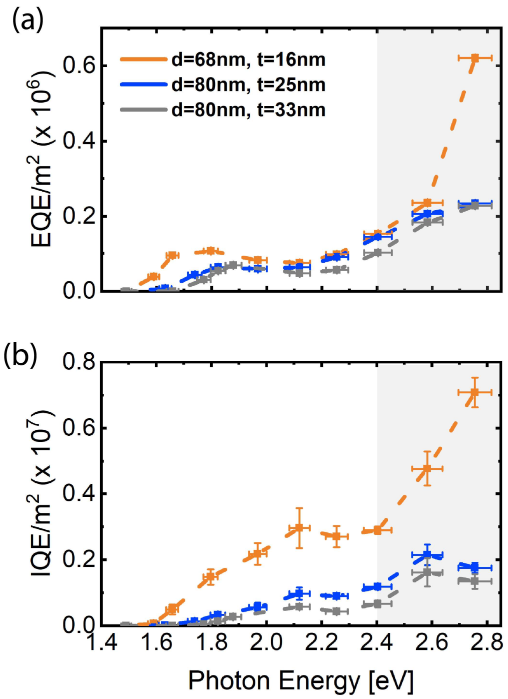

Figure S12. Normalized EQE and IQE spectra by the side and top surface of the NDs having different sizes. The gray shaded areas depict the purely interband region and the dashed lines are a guide to the eye

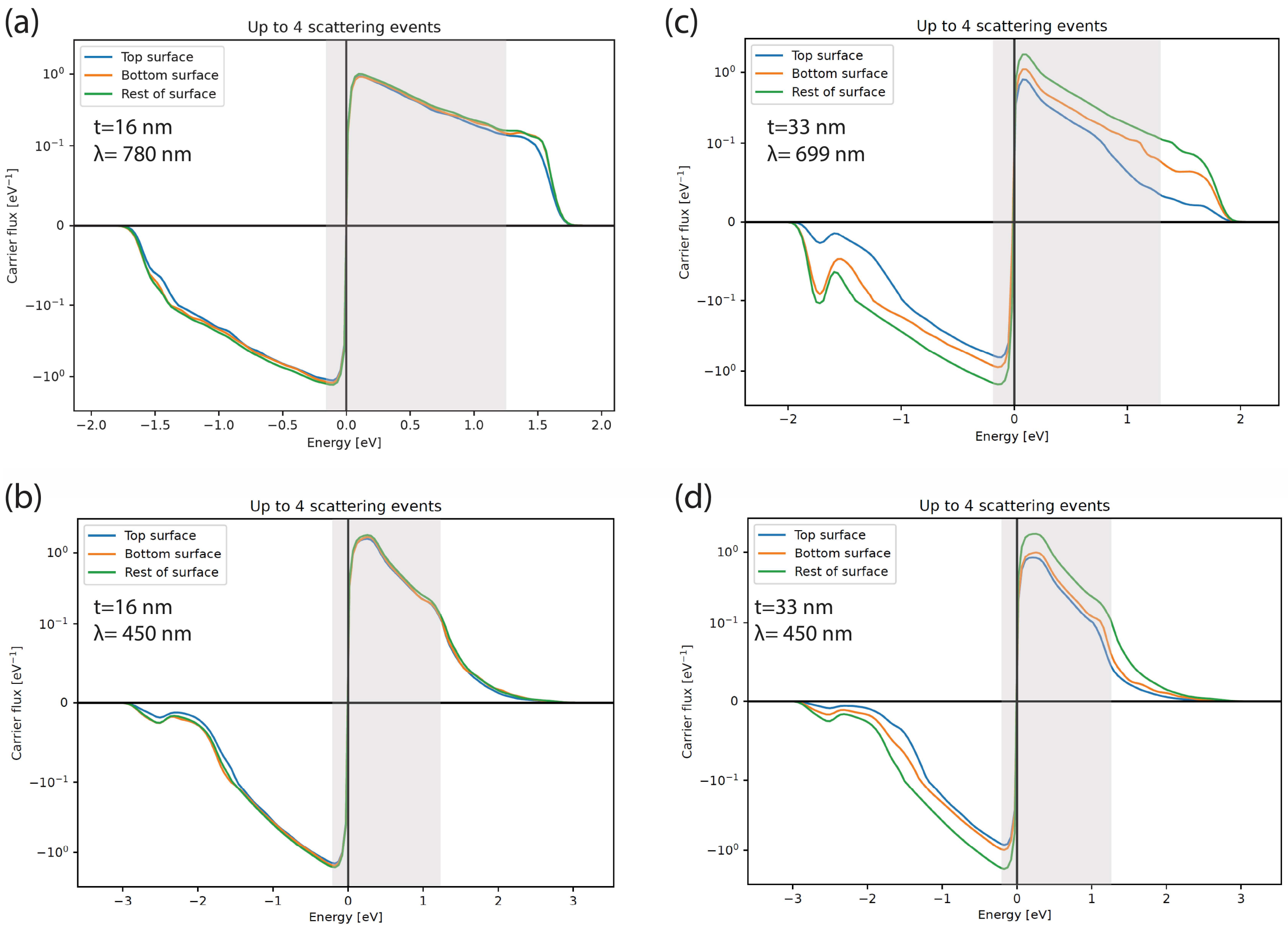

Figure S13. Calculated energy-resolved carrier fluxes reaching the top, bottom, and side surfaces of Au NDs having thicknesses of 16 and 33 nm after 4 scattering events, under illumination at their resonance wavelength (a and c) and 450 nm (b and d). The carrier energies are referenced to the Au Fermi level located at 0 eV. Negative values of hot-carrier energy correspond to hot holes (left side) and positive values correspond to hot electrons (right side). The shaded area shows the position of the reaction barrier (-0.19 eV) and the Schottky barrier (1.25 eV).

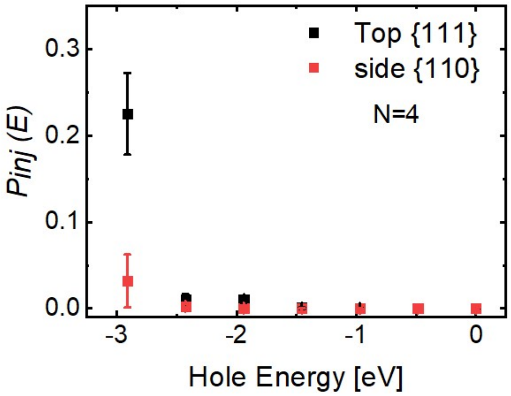

Figure S14. Estimated injection probability (P_{inj}) for hot holes collected from the top {111} and side {110} facets after four scattering events (N=4).

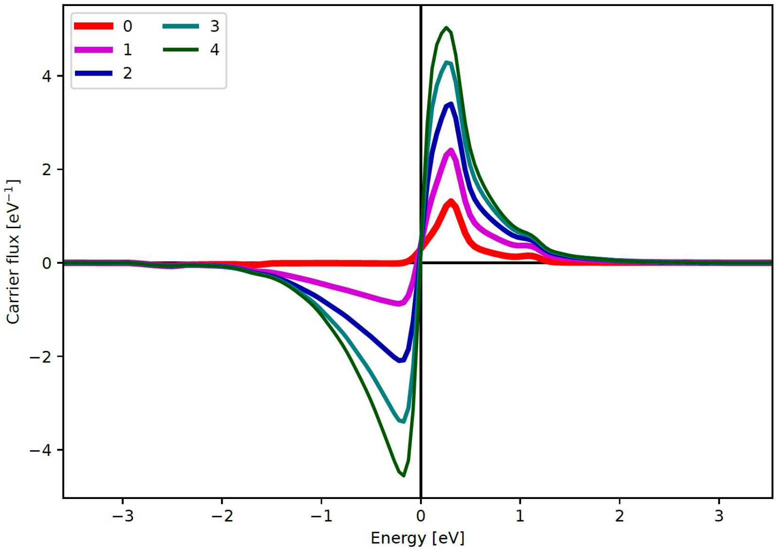

Figure S15. Calculated energy-resolved carrier fluxes reaching the surfaces of 16 nm-thick Au NDs directly (N=0) or upon scattering (N=1 to 4) under illumination at 450 nm (2.75 eV), showing the flux distribution of initial and homogenized carriers is the same at energies below -2 eV and above 2 eV.

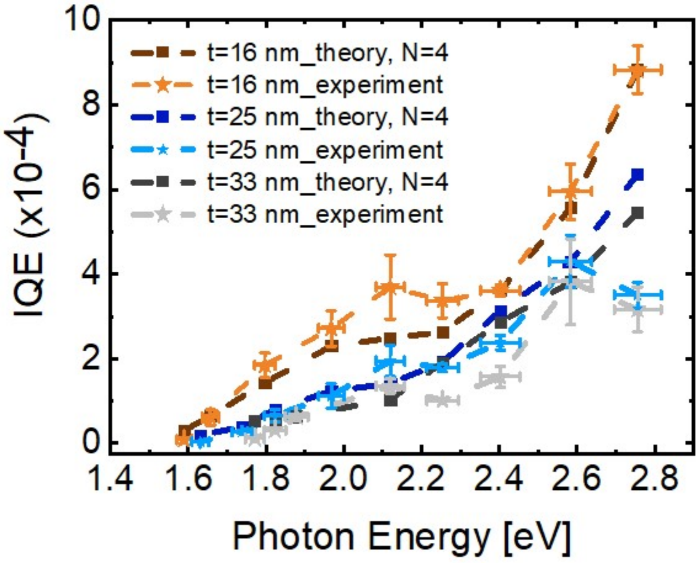

Figure S16. Calculated IQE spectra based on energy-resolved hot hole fluxes collected from the top and side surfaces and estimated P_{inj} in Figure S12 for scattered carriers (N=4) together with the experimentally determined IQEs for 16, 25 and 33 nm-thick Au ND heterostructures. The dashed lines are a guide to the eye.

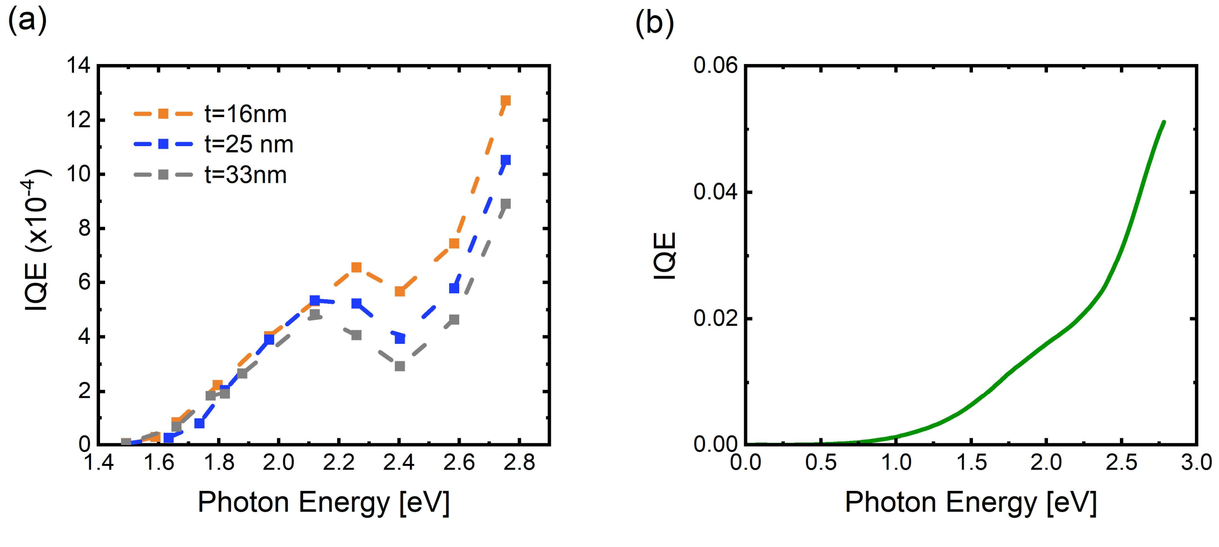

Figure S17. Calculated IQE spectra (a) based on the energy-resolved hot hole fluxes collected from the bottom and side surfaces and (b) based on the initial generation probability of the hot carrier in Au from ref [7] and estimated P_{inj} in **Figure 4.d** for non-scattered carriers (N=0). The dashed lines in panel (a) are a guide to the eye.

To compare the experimentally IQE obtained from our solid-state photocurrent measurements with our model, the same transport simulation was employed considering the Schottky barrier height of 1.25 eV across the Au/TiO_2 interface and assuming tangential momentum conservation for estimating the $P_{inj}(E)$. The predicted IQE for the hot electrons that reach the bottom interface directly or via scattering (up to 4 scattering events) for the 14 nm-thick stripe heterostructure is plotted in **Figure S18**, exhibiting the closest agreement with the experimental data (**Figure 3.h**) only for the no scattering condition. The computed IQE spectrum up to 4 scattering events shows a huge deviation from the ballistic condition, in particular after 2.2 eV. This result indicates that the majority of hot electrons undergo no scattering events before being collected at Au/TiO_2 interface in our device, consistent with the previous solid-state study on Au/n-GaN photodiode.[18] In fact, occurrence of the largest carrier generation at this interface and the suitable design of our nanoantenna thickness comparable with the average mean-free path of hot electrons (~10-40 nm up to 2 eV[7,19]) favor ballistic collection.

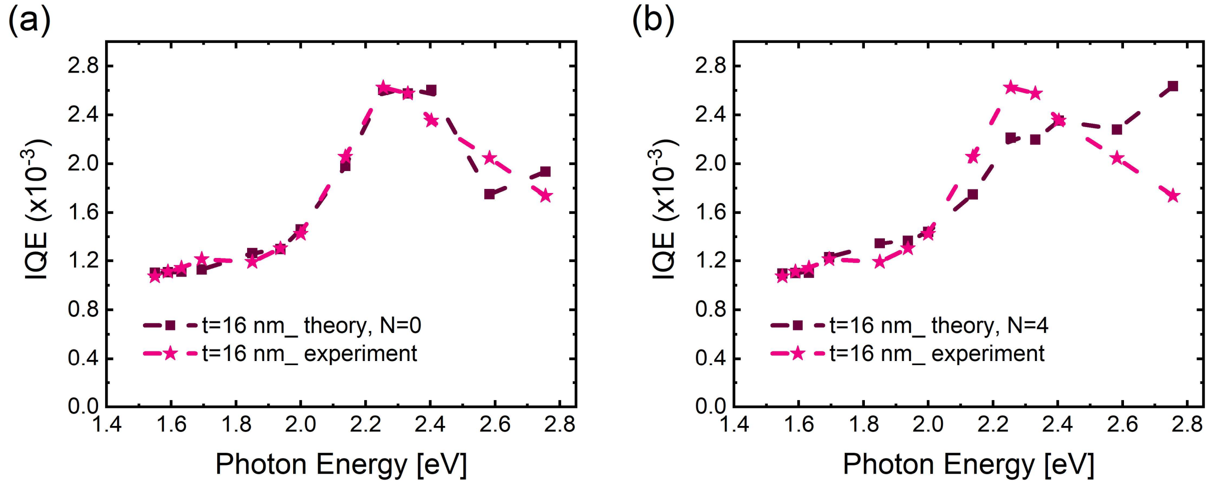

Figure S18. Calculated IQE spectrum based on energy-resolved hot electron fluxes for the (a) ballistic (N=0) and (b) scattering conditions (N= 4) and estimated injection probability under the assumption of tangential momentum conservation and Schottky barrier of 1.25 eV at Au/TiO_2 heterojunction. The dashed lines are a guide to the eye.

References

(1) Kiani, F.; Tagliabue, G. High Aspect Ratio Au Microflakes via Gap-Assisted Synthesis. *Chem. Mater.* **2022**, *34* (3), 1278–1288.

(2) Jiao, L.; Fan, B.; Xian, X.; Wu, Z.; Zhang, J.; Liu, Z. Creation of Nanostructures with Poly(Methyl Methacrylate)-Mediated Nanotransfer Printing. *J. Am. Chem. Soc.* **2008**, *130* (38), 12612–12613.

(3) Olmon, R. L.; Slovick, B.; Johnson, T. W.; Shelton, D.; Oh, S.-H.; Boreman, G. D.; Raschke, M. B. Optical Dielectric Function of Gold. *Phys. Rev. B* **2012**, *86* (23), 235147.

(4) Siefke, T.; Kroker, S.; Pfeiffer, K.; Puffky, O.; Dietrich, K.; Franta, D.; Ohlídal, I.; Szeghalmi, A.; Kley, E.-B.; Tünnermann, A. Materials Pushing the Application Limits of Wire Grid Polarizers Further into the Deep Ultraviolet Spectral Range. *Adv. Opt. Mater.* **2016**, *4* (11), 1780–1786.

(5) König, T. A. F.; Ledin, P. A.; Kerszulis, J.; Mahmoud, Mahmoud. A.; El-Sayed, M. A.; Reynolds, J. R.; Tsukruk, V. V. Electrically Tunable Plasmonic Behavior of Nanocube–Polymer Nanomaterials Induced by a Redox-Active Electrochromic Polymer. *ACS Nano* **2014**, *8* (6), 6182–6192.

(6) Jermyn, A. S.; Tagliabue, G.; Atwater, H. A.; Goddard, W. A.; Narang, P.; Sundararaman, R. Transport of Hot Carriers in Plasmonic Nanostructures. *Phys. Rev. Mater.* **2019**, *3* (7), 075201.

(7) Brown, A. M.; Sundararaman, R.; Narang, P.; Goddard, W. A. I.; Atwater, H. A. Nonradiative Plasmon Decay and Hot Carrier Dynamics: Effects of Phonons, Surfaces, and Geometry. *ACS Nano* **2016**, *10* (1), 957–966.

(8) Yu, Y.; Sundaresan, V.; Willets, K. A. Hot Carriers versus Thermal Effects: Resolving the Enhancement Mechanisms for Plasmon-Mediated Photoelectrochemical Reactions. *J. Phys. Chem. C* **2018**, *122* (9), 5040–5048.

(9) Yu, Y.; Williams, J. D.; Willets, K. A. Quantifying Photothermal Heating at Plasmonic Nanoparticles by Scanning Electrochemical Microscopy. *Faraday Discuss.* **2018**, *210* (0), 29–39.

(10) Sun, C.-K.; Vallée, F.; Acioli, L. H.; Ippen, E. P.; Fujimoto, J. G. Femtosecond-Tunable Measurement of Electron Thermalization in Gold. *Phys. Rev. B* **1994**, *50* (20), 15337–15348.

(11) Schoenlein, R. W.; Lin, W. Z.; Fujimoto, J. G.; Eesley, G. L. Femtosecond Studies of Nonequilibrium Electronic Processes in Metals. *Phys. Rev. Lett.* **1987**, *58* (16), 1680–1683.

(12) Zavelani-Rossi, M.; Polli, D.; Kochtcheev, S.; Baudrion, A.-L.; Béal, J.; Kumar, V.; Molotokaite, E.; Marangoni, M.; Longhi, S.; Cerullo, G.; Adam, P.-M.; Della Valle, G. Transient Optical Response of a Single Gold Nanoantenna: The Role of Plasmon Detuning. *ACS Photonics* **2015**, *2* (4), 521–529.

(13) Baffou, G.; Berto, P.; Bermúdez Ureña, E.; Quidant, R.; Monneret, S.; Polleux, J.; Rigneault, H. Photoinduced Heating of Nanoparticle Arrays. *ACS Nano* **2013**, *7* (8), 6478–6488.

(14) Naef, A.; Mohammadi, E.; Tsoulos, T. V.; Tagliabue, G. Light-Driven Thermo-Optical Effects in Nanoresonator Arrays. *Adv. Opt. Mater.* **2023**, 2300698. https://doi.org/10.1002/adom.202300698.

(15) Konopka, S. J.; McDuffie, Bruce. Diffusion Coefficients of Ferri- and Ferrocyanide Ions in Aqueous Media, Using Twin-Electrode Thin-Layer Electrochemistry. *Anal. Chem.* **1970**, *42* (14), 1741–1746.

(16) Yu, Y.; Wijesekara, K. D.; Xi, X.; Willets, K. A. Quantifying Wavelength-Dependent Plasmonic Hot Carrier Energy Distributions at Metal/Semiconductor Interfaces. *ACS Nano* **2019**, *13* (3), 3629–3637.

(17) Bard, A. J.; Mirkin, M. V. *Scanning Electrochemical Microscopy, Second Edition*; CRC Press, **2012**.

(18) Tagliabue, G.; Jermyn, A. S.; Sundararaman, R.; Welch, A. J.; DuChene, J. S.; Pala, R.; Davoyan, A. R.; Narang, P.; Atwater, H. A. Quantifying the Role of Surface Plasmon Excitation and Hot Carrier Transport in Plasmonic Devices. *Nat. Commun.* **2018**, *9* (1), 1–8.

(19) Bernardi, M.; Mustafa, J.; Neaton, J. B.; Louie, S. G. Theory and Computation of Hot Carriers Generated by Surface Plasmon Polaritons in Noble Metals. *Nat. Commun.* **2015**, *6* (1), 7044.
